# Associations of sugar intake, high-sugar dietary pattern, and the risk of dementia: a prospective cohort study of 210,832 participants

**DOI:** 10.1186/s12916-024-03525-6

**Published:** 2024-07-18

**Authors:** Sirui Zhang, Yi Xiao, Yangfan Cheng, Yuanzheng Ma, Jiyong Liu, Chunyu Li, Huifang Shang

**Affiliations:** 1 Department of Neurology, Laboratory of Neurodegenerative Disorders, Rare Disease Center, West China Hospital, Sichuan University, Chengdu, 610041 China; 2 Laboratory of Neurodegenerative Disorders, National Clinical Research Center for Geriatric, West China Hospital, Sichuan University, Chengdu, 610041 China; 3 School of Medicine, West China Hospital, Sichuan University, Chengdu, 610041 China

**Keywords:** Dementia, Alzheimer’s disease, Sugar intake, Dietary pattern

## Abstract

**Background:**

Limited evidence demonstrated the potential relationship between dietary sugar intake and dementia. This association demands further clarification in a large-scale population.

**Methods:**

A total of 210,832 participants from the UK Biobank cohort were included in this prospective cohort study. Absolute and relative sugar intake and high-sugar dietary scores were utilized to reflect dietary sugar intake. Absolute sugar intake was identified by the Oxford WebQ in the UK Biobank. Relative sugar intake was calculated by dividing the absolute sugar intake by total diet energy. High-sugar dietary pattern was identified using the method of reduced rank regression. Cox proportional hazards regression analyses and restricted cubic splines were performed to examine the longitudinal associations between dietary sugar intake and all-cause dementia and its main subtype, Alzheimer’s disease. Explorative mediation analyses were conducted to explore underlying mechanisms.

**Results:**

Increased absolute sugar intake (g/day) was significantly associated with a higher risk of all-cause dementia (HR = 1.003, [95%CI: 1.002–1.004], *p* < 0.001) and Alzheimer’s disease (1.002, [1.001–1.004], 0.005). Relative sugar intake (%g/kJ/day) also demonstrated significant associations with all-cause dementia (1.317, [1.173–1.480], *p* < 0.001) and Alzheimer’s disease (1.249, [1.041–1.500], 0.017), while the high-sugar dietary score was only significantly associated with a higher risk of all-cause dementia (1.090, [1.045–1.136], *p* < 0.001). In addition, both sugar intake and high-sugar dietary score demonstrated significant non-linear relationships with all-cause dementia and Alzheimer’s disease (all *p* values for non-linearity < 0.05).

**Conclusions:**

Our study provided evidence that excessive sugar intake was associated with dementia. Controlling the excess consumption of dietary sugar may be of great public health implications for preventing dementia.

**Supplementary Information:**

The online version contains supplementary material available at 10.1186/s12916-024-03525-6.

## Background

Dementia is an important and serious public health challenge with a rapidly increasing incidence rate due to the acceleration of the aging process worldwide [1]. The Global Burden of Disease Study 2019 reported that dementia patients would increase from 57.4 million cases globally in 2019 to 152.8 million cases in 2050 [2]. Treatments targeting amyloid-related pathologies at an early stage have made important advances recently and provided hope for patients albeit with challenges [3, 4]. However, taking effective measures to prevent the onset of dementia is still a crucial and cost-effective choice.

Numerous nutritional epidemiological studies have emphasized the important role of diet intervention in the prevention of dementia [5, 6]. As excess sugar intake was validated to be associated with cardiovascular diseases [7, 8], metabolic disturbances [7, 9], and systemic inflammation [10], which might contribute to increasing the risk for dementia [11–14], we reasonably hypothesized that high sugar intake was an important modifiable lifestyle risk factor of dementia. Prior studies predominantly focused on the effect of some specific sugar sources, such as sugar-sweetened beverages (SSBs), on the incidence of dementia [15]. Sugar intake from various other sources in daily diet is also worthy of attention. Nevertheless, only a few studies evaluated the role of dietary sugar intake on dementia [16, 17].

Accordingly, the current study aimed to examine the associations between sugar intake, high-sugar dietary pattern, and the risk of dementia in a large general population and explore the underlying mechanisms by taking indicators of vascular disorders, metabolic abnormalities, and systemic inflammation into account in the mediation analyses.

## Methods

### Data source

The data analyzed in the current study were obtained from the UK Biobank database, which is a large-scale population-based prospective cohort [18]. Written informed consent forms from all participants were obtained and ethnic approval was granted by the UK North West Multi-Centre Research Ethics Committee (no. 21/NW/0157). The detailed introduction of the database was described on the UK Biobank website (http://www.ukbiobank.ac.uk/resources/).

### Study populations

**Fig. 1:**
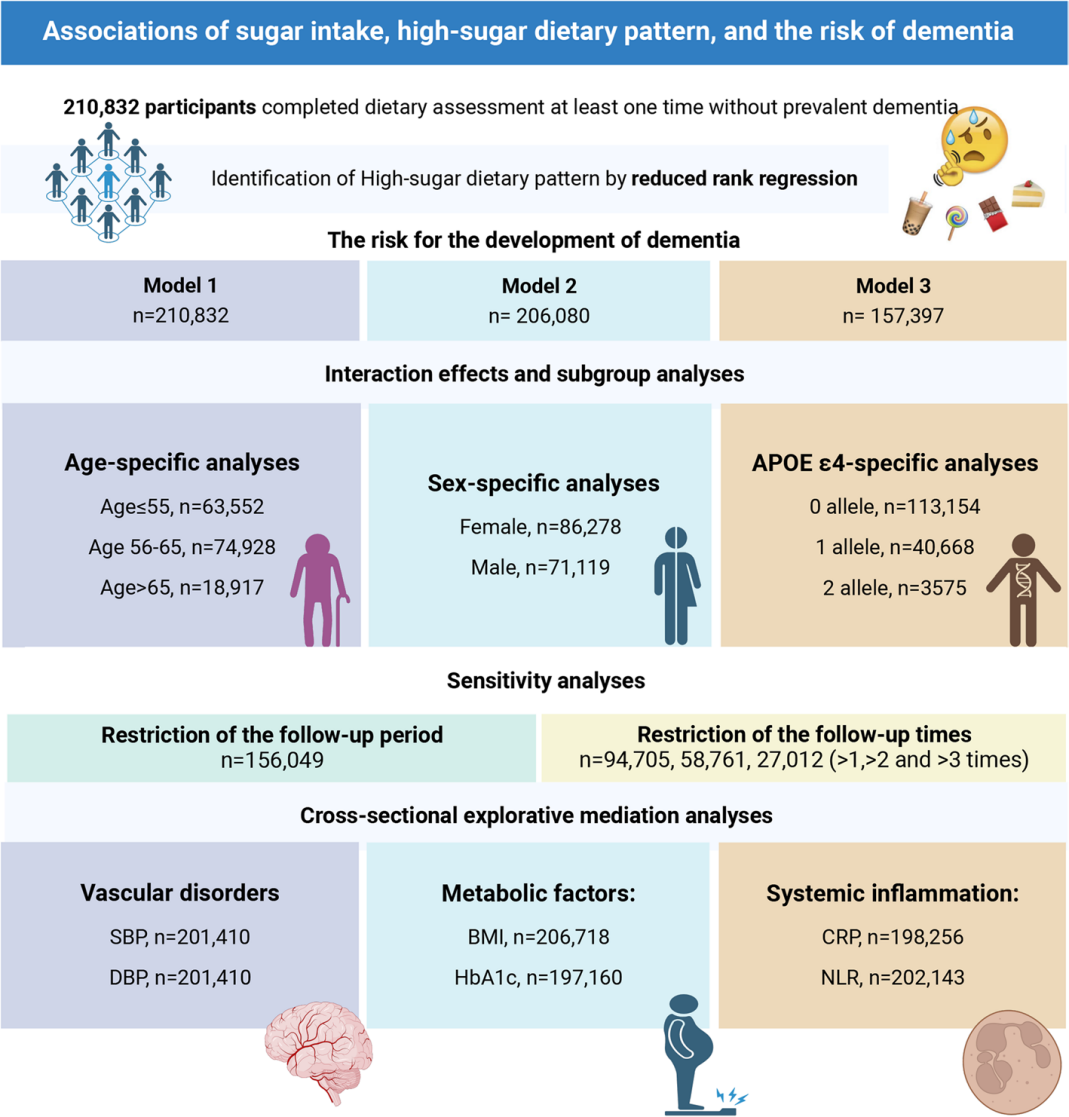
Flowchart of the study design and the number of available participants

Among the initial 502,382 participants, 210,954 individuals who completed at least one dietary questionnaire, based on a 24-h dietary recall of the previous day, were included in the current study. Participants who were recorded as dementia patients before attending the assessment of the dietary questionnaire were excluded (*n* = 122). The ascertainment of dementia outcomes in the present study will be introduced in the following section. In total, 210,832 individuals were included in the analysis. The flowchart of the study design and the number of available participants is illustrated in Fig. 1.

### Sugar intake measurement

The term “sugar” refers to monosaccharides and disaccharides, including fructose, glucose, sucrose, maltose, galactose, and lactose according to its chemical classification [19]. UK Biobank applied an automated web-based 24-h dietary questionnaire named the Oxford WebQ to collect information about participants’ consumption of food and beverages during the last 24 h [20]. The quantity for each consumed food or beverage item was determined by multiplying its assigned portion size by the consumed amount, and then the built-in algorithms of the system would evaluate the food composition data for each participant and calculate the intakes of specific nutrients (such as total sugars), as described detailed in the previous literature [21, 22]. The web-based Oxford WebQ has been validated against an interviewer-administered 24-h dietary recall [20] and also performed well across key nutrients including protein, potassium, sugar intake, and total energy when using objective biomarkers as the standard [23]. Absolute total sugar (unit: g/day) and its subtypes (fructose, glucose, sucrose, maltose, lactose, and other sugars) data were collected from the UK Biobank and analyzed in the current study. Relative sugar intake (unit: %g/kJ/day) was defined as absolute sugar intake divided by total energy intake (unit: kJ) in the present study. We also calculated the quartile of the daily intake of the different types of sugars and categorized the participants into four groups: Q1(lowest intake), Q2, Q3, and Q4 (highest intake). The first dietary assessment was performed at the assessment center from April 2009 to September 2010. Four additional questionnaire rounds were conducted online, with invitations being emailed to participants at 3–4 monthly intervals between April 2009 and June 2012. The mean values of the exposures from the available data were calculated in the case that participants attended more than one assessment to reflect a long-term exposed state.

### Identification of high-sugar dietary pattern

Reduced rank regression (RRR) is a widely used method to identify the specific dietary pattern in the study of nutritional epidemiology and the principle of this method has been introduced in previous literature [24]. In the present study, RRR was utilized to derive a high-sugar dietary pattern that could explain the maximum variation in sugar intake (response variables) according to the consumption of different food groups (exposure variables). Relative fructose, glucose, sucrose, maltose, lactose, and other sugar intake were set as response variables. Fifty-one food groups based on food intake data from the Oxford WebQ questionnaire were set as exposure variables, as presented in Additional file 1: Table S1. Faction loading for each food group was calculated based on the linear function to reflect the estimate of the association between food group consumption and specific nutrient consumption. High-sugar dietary pattern score for each participant was calculated by multiplying the standardized intake of each food group by its factor loading, representing the extent to which the participant’s dietary pattern reflected the high-sugar dietary pattern relative to that of other participants. Individuals who consumed more food groups with a positive factor loading would obtain higher scores. The dietary pattern that explained the maximum variation in the sugar intake was retained for the subsequent analyses, in consistency with previous reports [25, 26]. RRR contributes to overcoming the limitation of only focusing on the role of one single type of nutrient, as the interactions of different food group combinations were considered in the analysis, but not merely the intake of specific nutrients.

### Dementia ascertainment

The primary outcome of the current study was all-cause dementia. We also investigated its major subtype, Alzheimer’s disease (AD), as the secondary outcome. The dementia incidents were ascertained through both algorithmically defined dementia outcomes and first occurrences data in the UK Biobank. Algorithmically defined dementia outcomes were derived from the algorithmic combinations of UK Biobank’s linked data from hospital admissions and death registries, along with self-reported medical conditions at baseline. First occurrences data additionally recorded the primary care data to supplement the algorithmically defined dementia outcomes. Since we excluded those participants diagnosed with all-cause dementia before baseline assessment, self-reported dementia patients were not included in the analysis to increase the accuracy of dementia outcomes ascertainment. The detailed description of the algorithmically defined outcomes and first occurrence of health outcomes in the UK Biobank can be found on the website (biobank.ndph.ox.ac.uk/ukb/ukb/docs/alg_outcome_main.pdf, and biobank.ndph.ox.ac.uk/ukb/ukb/docs/first_occurrences_outcomes.pdf). The International Classification of Diseases (ICD)-9 and ICD-10 coding systems were used to record the diagnoses of dementia in the UK Biobank, as shown in Additional file 1: Table S2. Previous studies have reported a positive predictive value of 82.5% regarding the all-cause dementia events using the UK routinely collected healthcare datasets when combining the data source of primary care, hospital admissions, and death registries, validating the accuracy of dementia diagnoses in the UK Biobank [27]. Follow-up started when the dietary questionnaire was completed for the first time and ended at the time of the earliest dementia incident (all-cause dementia and AD), death, loss to follow-up, or latest data update (February 2022), whichever occurred first.

### Covariates

The covariates were selected based on the identified risk factors for dementia in previous reports [28–30], including sex, age at baseline, ethnicity, body mass index (BMI), education level, Townsend Deprivation Index (TDI), smoking status, drinking status, metabolic equivalent of tasks (MET), diabetes, hypertension, and apolipoprotein E (APOE) ε4 status. Ethnicity was categorized as white and non-white. BMI was categorized as less than 18.5, 18.5–24.9, 25.0–29.9, and ≥ 30 (unit: kg/m^2^) according to the World Health Organization’s criteria. Education level was categorized as higher education (college degree/university degree/other professional qualification) and lower education. TDI reflected an area-based socioeconomic status and was divided into quartiles in the analyses. Smoking and drinking status were classified into three groups: never, previous, and current. MET was an indicator of total physical activity (unit: minutes/week) and was divided into quartiles. The number of APOE ε4 alleles was calculated based on two single-nucleotide polymorphisms (rs7412 and rs429358) and was classified into three groups in the current study according to the number of carriers: 0, 1, and 2. All the variables used in the current study are listed in Additional file 1: Table S3.

### Statistical analysis

To summarize the baseline characteristics of the populations according to total sugar intake, continuous variables were described as mean (standard deviation, SD) and categorical variables were described in the form of frequency (percentage).

Cox proportional hazard regression models were used to investigate the longitudinal association between sugar exposures (absolute sugar intake, relative sugar intake, and high-sugar dietary score) and dementia incidents (all-cause dementia and AD). Continuous variables were transformed into categorical variables by their quartile distributions from low to high (Q1 to Q4) and the Q1 group was set as the reference group for analyses. Model 1 was unadjusted. Model 2 was adjusted for baseline demographic information and socio-economic factors including sex, age at baseline, ethnicity, education level, BMI, and TDI. Model 3 was further adjusted for important lifestyle factors, comorbidities, and genotype factor including smoking status, drinking status, metabolic equivalent of tasks (MET), diabetes, hypertension, and apolipoprotein E (APOE) ε4 status. Participants with missing covariates information were excluded from the corresponding analyses. The complete information regarding missing covariants is listed in Additional file 1: Table S4. The proportional hazards assumptions were tested using the method of Schoenfeld residuals and we observed no violation of the assumption. The potential non-linear associations between sugar intake and dementia incidents were also tested in Model 3 using the method of restricted cubic spline (RCS), with the reference value set at the median, and four knots set at the 25th, 50th, 75th, and 95th centiles. Hazard ratios (HRs) and 95% confidence intervals (CIs) were calculated. *p* values for the trend were calculated using the median of each quartile group as a quasi-continuous variable in the model.

We then examined the potential interaction effects of age, sex, BMI, APOE ε4 status, diabetes, and hypertension with sugar intake on the risk of dementia by adding multiplicative interaction terms in the Cox proportional hazard model and performed subgroup analyses further. Regarding sensitivity analyses, we first excluded participants who developed dementia within 3 years of follow-up to minimize the effect of reverse causation. Additionally, we repeated previously mentioned analyses among participants who completed at least two, three, and four times of dietary assessments which might reflect a more long-term and stable dietary preference.

Cardiovascular factors, systemic inflammation, and metabolic disturbance might contribute to the effect of sugar intake on the risk of dementia incidents. In the exploratory mediation analyses, we evaluated the mediation effects of inflammatory markers including neutrophil–lymphocyte ratio and C-reactive protein (CRP), metabolic markers including BMI and HbA1c, and vascular markers including systolic blood pressure (SBP) and diastolic blood pressure (DBP) on the association between total sugars intake and dementia cross-sectionally. The “Lavaan” package in R software was applied to perform mediation analyses with a nonparametric Bootstrapping test with 1000 iterations.

We used STATA software (version: 17.0) to perform RRR analysis^24^ and R software (version: 4.2.0) for all other analyses. A two-sided *p* value of < 0.05 was accepted as statistically significant.

## Results

### Population characteristics

The population characteristics were summarized according to the quartile distribution of absolute daily sugar intake, as presented in Table 1. A total of 210,832 participants without all-cause dementia at the baseline visit were enrolled in the current study. The mean age of the included populations was 56.08 ± 7.99 years old, and 116,153 (55.09%) were females. Participants with high intake of total sugars appeared to be older (Table 1). The mean age of the participants with the lowest quartile of total sugar intake was 55.35 ± 7.93 years, while that of the participants with the highest quartile was 56.45 ± 8.08 years. Additionally, males, current smokers and drinkers, and participants with vascular diseases were more likely to consume more sugar. Finally, 1877 cases of all-cause dementia and 781 cases of AD occurred during the mean follow-up period of 11.80 ± 1.66 years.

**Table 1:** Baseline characteristics of study participants by the quartile of absolute total sugar intake

Characteristics	Absolute total sugar intake				
	**Total**	**Q1**	**Q2**	**Q3**	**Q4**
Number	210,832	52,694	52,726	52,705	52,707
Sex					
Female	116,153 (55.09)	32,741 (62.13)	30,890 (58.59)	28,391 (53.87)	24,131 (45.78)
Male	94,679 (44.91)	19,953 (37.87)	21,836 (41.41)	24,314 (46.13)	28,576 (54.22)
Age at baseline, years	56.08 (7.99)	55.35 (7.93)	56.14 (7.85)	56.38 (7.89)	56.45 (8.08)
Ethnicity					
White	198,110 (95.46)	49,442 (95.35)	49,549 (95.54)	49,581 (95.51)	49,538 (95.47)
Non-white	9413 (4.54)	2413 (4.65)	2314 (4.46)	2333 (4.49)	2353 (4.53)
Smoking status					
Current	21,722	5435 (10.48)	5399 (10.42)	5375 (10.37)	5513 (10.63)
Never	113,712	28,524 (54.84)	28,403 (54.83)	28,544 (55.04)	28,241 (54.46)
Previous	71,934	17,885 (34.69)	18,004 (34.75)	17,938 (34.59)	18,107 (34.91)
Alcohol consumption					
Current	191,300 (92.00)	47,771 (91.93)	47,798 (91.96)	47,873 (92.05)	47,858 (92.05)
Never	9185 (4.42)	2256 (4.34)	2332 (4.49)	2305 (4.43)	2292 (4.41)
Previous	7453 (3.58)	1936 (3.73)	1846 (3.55)	1829 (3.52)	1842 (3.54)
Higher education					
Yes	117,155 (55.59)	27,577 (52.36)	30,007 (56.93)	30,577 (58.04)	28,994 (55.03)
No	93,593 (44.41)	25,096 (47.64)	22,701 (43.07)	22,106 (41.96)	23,690 (44.97)
Townsend deprivation index	− 1.30 (3.09)	− 1.28 (3.11)	− 1.30 (3.09)	− 1.31 (3.08)	23,691
Townsend deprivation index quartile					
Q1 (least deprived)	52,302 (25.16)	13,019 (25.05)	13,096 (25.21)	13,086 (25.17)	13,101 (25.20)
Q2	51,737 (24.88)	12,995 (25.01)	12,921 (24.87)	12,950 (24.90)	12,871 (24.75)
Q3	51,938 (24.99)	12,807 (24.64)	13,010 (25.05)	13,025 (25.05)	13,116 (25.23)
Q4 (most deprived)	51,911 (24.97)	13,148 (25.30)	12,919 (24.87)	12,938 (24.88)	12,906 (24.82)
Diabetes					
No	201,794 (95.73)	49,443 (93.86)	50,397 (95.60)	50,925 (96.64)	51,029 (96.83)
Yes	8994 (4.27)	3237 (6.14)	2320 (4.40)	1768 (3.36)	1669 (3.17)
Vascular diseases					
No	154,773 (73.53)	38,203 (72.62)	38,920 (73.93)	39,043 (74.20)	38,607 (73.38)
Yes	55,710 (26.47)	14,403 (27.38)	13,725 (26.07)	13,579 (25.80)	14,003 (26.62)
BMI, kg/m2	26.96 (4.66)	27.36 (4.90)	26.87 (4.60)	26.70 (4.50)	26.90 (4.60)
BMI groups					
< 18.5	1095 (0.53)	253 (0.49)	277 (0.54)	262 (0.51)	303 (0.59)
≥ 18·5 to < 25·0	67,278 (32.49)	16,709 (32.29)	16,730 (32.33)	16,887 (32.61)	16,952 (32.73)
≥ 25·0 to < 30·0	88,224 (42.61)	22,083 (42.67)	22,007 (42.53)	22,083 (42.64)	22,051 (42.58)
≥ 30·0	50,465 (24.37)	12,703 (24.55)	12,726 (24.60)	12,554 (24.24)	12,482 (24.10)
MET min/week	2647.67 (2705.92)	2644.57 (2700.75)	2648.76 (2719.54)	2655.88 (2703.30)	2641.44 (2700.1)
MET quartile					
1	42,065 (24.94)	10,489 (24.88)	10,572 (25.07)	10,566 (25.00)	10,438 (24.81)
2	42,268 (25.06)	10,630 (25.22)	10,574 (25.07)	10,502 (24.85)	10,562 (25.10)
3	42,235 (25.04)	10,507 (24.93)	10,483 (24.86)	10,550 (24.96)	10,695 (25.42)
4	42,015 (24.96)	10,525 (24.97)	10,544 (25.00)	10,652 (25.20)	10,384 (24.68)
APOE ε4					
0	143,338 (71.94)	36,035 (72.58)	35,872 (71.97)	35,908 (72.05)	35,523 (71.13)
1	51,419 (25.81)	12,536 (25.25)	12,866 (25.81)	12,785 (25.65)	13,232 (2.65)
2	4501 (2.26)	1075 (2.17)	1105 (2.22)	1142 (2.29)	1179 (2.36)

Data is presented as mean (SD) for continuous variables and as frequency (percentage) for categorical variables

*BMI* body mass index, *MET* metabolic equivalent of tasks, *APOE ε4* apolipoprotein E4

### Identification of high-sugar dietary pattern

**Fig. 2:**
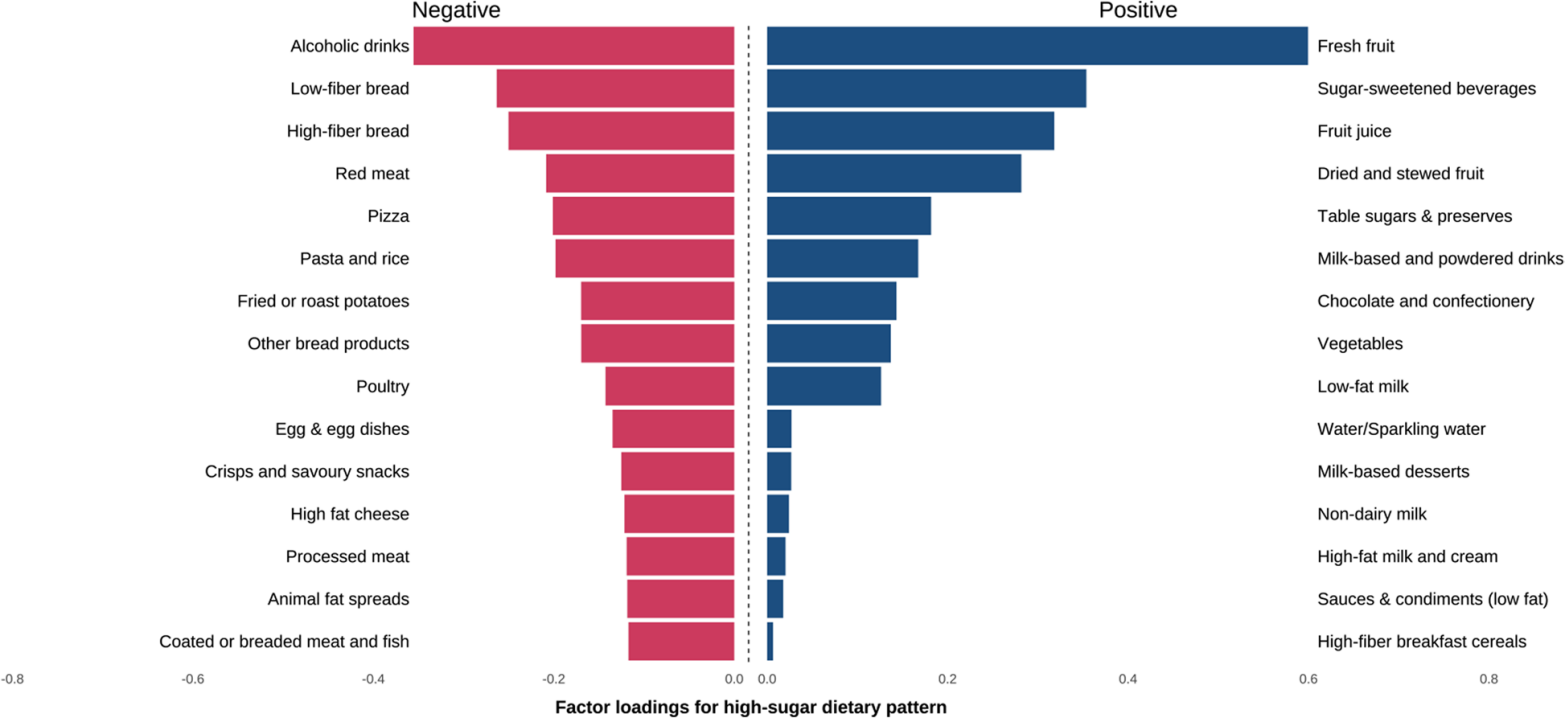
Factor loadings for high-sugar dietary pattern. Note: Only the top 15 food groups with negative factor loadings are preserved in the figure and the comprehensive information is listed in Supplementary Table 4

We identified the high-sugar dietary pattern that explained the maximum variation in the response variables. The high-sugar dietary pattern explained 28.2% of the variations in the six subtypes of sugar intake on average. This dietary pattern was characterized by high consumption of fresh fruit, sugar-sweetened beverages and other sugary drinks, fruit juice, dried and stewed fruit, table sugars and preserves, milk-based and powdered drinks, chocolate, and confectionery, as illustrated in Fig. 2. The mean absolute sugar intake increased from 102.976 g/day among individuals in quantile 1 (Q1) of high-sugar dietary scores to 162.243 g/day in Q4. Detailed information on factor loadings of different food groups and all types of sugar intake according to the quartile of high-sugar dietary scores is listed in Additional file 1: Table S5 and S6.

### Sugar intake and dementia

In the crude model 1, higher absolute and relative total sugar intake and high-sugar dietary scores demonstrated significant positive associations with the increased risk of all-cause dementia and AD. In the fully adjusted model 3, absolute and relative total sugar intake and high-sugar dietary scores remained statistically significant associations with all-cause dementia incidents. The corresponding HRs and 95%CI were 1.003 [1.002–1.004], 1.317 [1.173–1.480], and 1.090 [1.045–1.136], respectively. After transforming continuous variables into categorical variables according to their quartiles, the highest quartile group (Q4) of absolute and relative total sugar intake and high-sugar dietary scores demonstrated significant associations with17.1%, 32.3%, and 25.5% increased risk of all-cause dementia, compared to the lowest quartile group (all *p* for trend < 0.05). Regarding AD, both absolute and relative sugar intake were significantly associated with a linearly increased risk of AD, but not high-sugar dietary scores (*p* = 0.056). The longitudinal associations between absolute sugar intake, relative sugar intake, and high-sugar dietary scores and dementia are listed in Table 2. The comprehensive results of the analyses based on the three models are listed in Additional file 1: Table S7 and S8.

**Table 2:** Associations between sugar intake, high-sugar dietary score, and dementia

	All-cause dementia			AD		
	Number of events = 1391			Number of events = 570		
	HR	95% CI	*p*-value	HR	95% CI	*p*-value
**Absolute total sugar intake (g/day)**	1.003	1.002**–**1.004	< 0.001^*^	1.002	1.001**–**1.004	0.005^*^
**Absolute total sugar intake, quartile**						
Q1 (74.134)	Reference			Reference		
Q2 (105.784))	0.849	0.724**–**0.995	0.043^*^	0.914	0.712**–**1.174	0.483
Q3 (133.527)	0.898	0.769**–**1.049	0.175	0.993	0.779**–**1.267	0.958
Q4 (177.590)	1.171	1.013**–**1.355	0.033^*^	1.191	0.944**–**1.503	0.140
*p* for trend	0.003^*^			0.058		
Relative total sugar intake (%g/kJ/day)	1.317	1.173**–**1.480	< 0.001^*^	1.249	1.041**–**1.500	0.017^*^
Relative total sugar intake, quartile						
Q1 (0.980)	Reference			Reference		
Q2 (1.300)	0.964	0.822**–**1.132	0.658	0.983	0.764**–**1.265	0.892
Q3 (1.563)	0.945	0.805**–**1.109	0.489	0.938	0.729**–**1.207	0.619
Q4 (1.957)	1.323	1.140**–**1.535	< 0.001^*^	1.283	1.014**–**1.624	0.038^*^
*p* for trend	< 0.001^*^			0.021^*^		
High-sugar dietary score	1.09	1.045**–**1.136	< 0.001^*^	1.067	0.998**–**1.140	0.056
High-sugar dietary score, quartile						
Q1 (− 1.369)	Reference			Reference		
Q2 (− 0.370)	0.914	0.778**–**1.074	0.274	0.871	0.674**–**1.125	0.290
Q3 (0.348)	0.964	0.822**–**1.132	0.657	0.97	0.756**–**1.246	0.813
Q4 (1.364)	1.255	1.078**–**1.462	0.003^*^	1.196	0.942**–**1.520	0.142
*p* for trend	< 0.001^*^			0.053		

Results of Cox-proportional hazards analyses in fully adjusted model 3

*AD* Alzheimer’s disease, *HR* hazard ratio, *95% CI* 95% confidence interval

^*^Statistically significant difference was found among the groups (*p* < 0.05)

We further performed the analyses to validate the role of different types of sugar on the risk of dementia. The results showed that sucrose was most robustly associated with all-cause dementia and AD. As the different roles of sugar subtypes were not the main concern of the current research, we did not conduct further analyses. The detailed results of analyses between various subtypes of sugar and incidents of dementia are summarized in Additional file 1: Table S9 and S10.

**Fig. 3:**
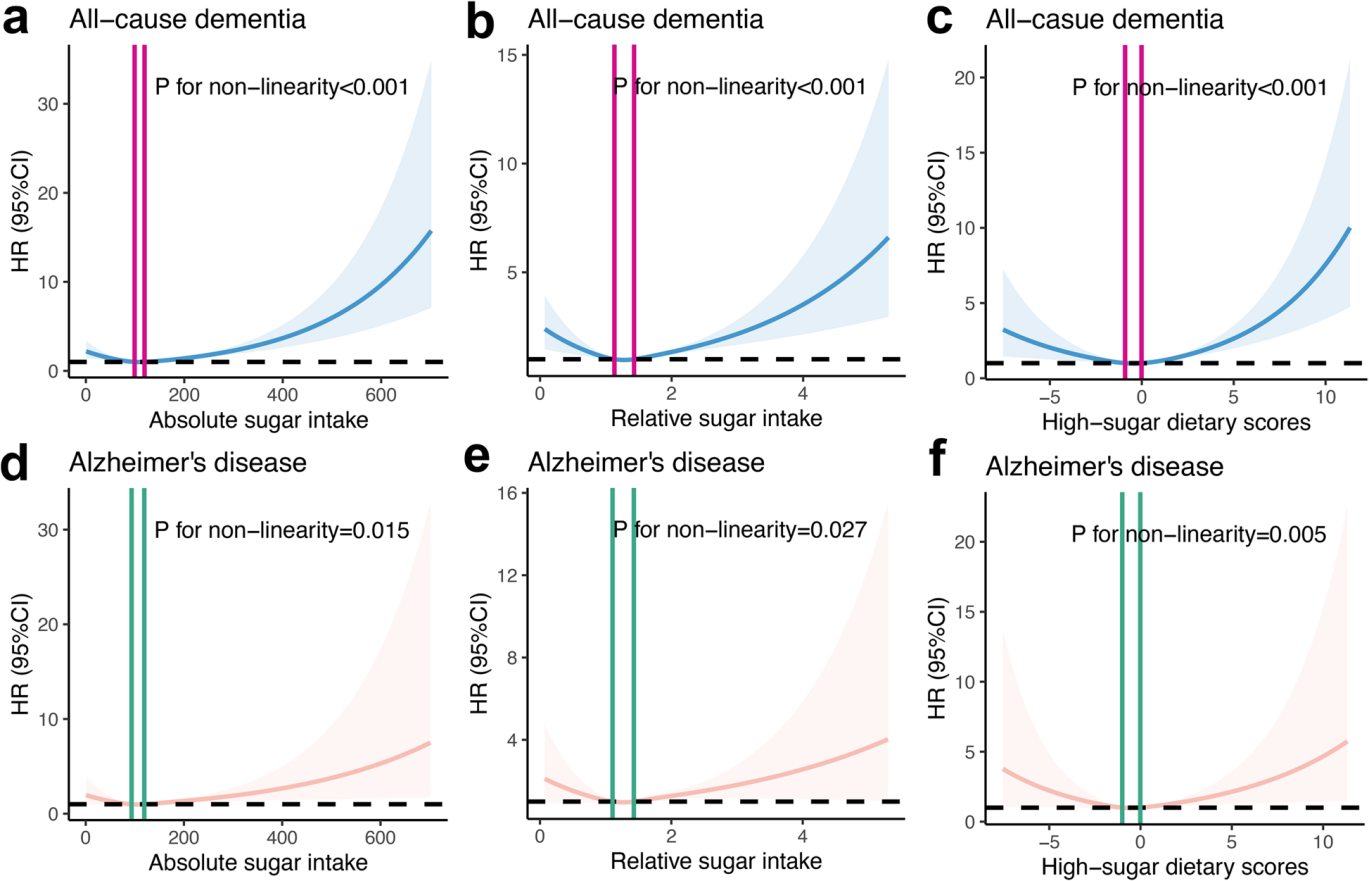
Restricted cubic spline models of the association between absolute sugar intake, relative sugar intake, and high-sugar dietary score and incident dementia. Note: The analyses were performed in fully adjusted model 3. The solid lines show hazard ratios (HRs) and the shaded areas show 95% confidential intervals (CIs). **a**-**c** Absolute sugar intake (g/day), relative sugar intake(%g/kJ/day), and high-sugar dietary score and the risk of all-cause dementia. **d**-**f** Absolute sugar intake (g/day), relative sugar intake(%g/kJ/day), and high-sugar dietary score and the risk of Alzheimer’s disease

We additionally performed RCS analyses to explore the potential non-linear associations between sugar intake and dementia. We observed significant non-linear associations between absolute and relative total sugar intake and high-sugar dietary scores and all-cause dementia and AD. They presented a similar pattern in which consumption of more than the median amount of sugar increased the risk of dementia more evidently, as shown in Fig. 3. The inflection points where HR = 1 are listed in Additional file 1: Table S11.

### Interaction effects and subgroup analyses

**Fig. 4:**
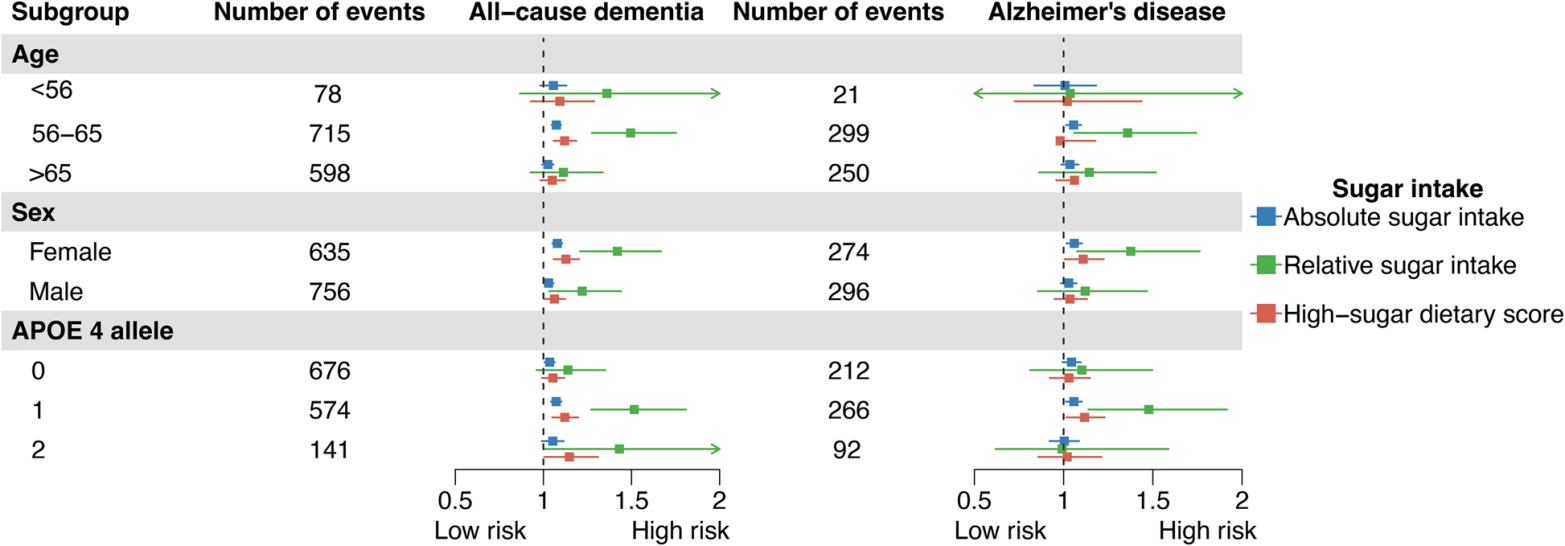
Associations between exposures and dementia in the age-specific, sex-specific, and APOE-specific subgroups. Note: For the clarity of presentation, a 20 g/day increment of absolute total sugar was considered as one unit of absolute total sugar in Fig. 4. The boxes show hazard ratios (HRs) and the lines show 95% confidential intervals (CIs). The analyses were performed in fully adjusted model 3

The results of interaction effects analyses are summarized comprehensively in Additional file 1: Table S12 and S13. Further, in the age-specific analyses, we found that the effect of sugar intake and high-sugar dietary pattern on all-cause dementia and AD only remained significant among middle-aged populations (56–65 years old), but not among young or aged people. In the sex-specific analyses, generally, the effect of sugar intake on dementia appeared to be more prominent among females. In males, absolute and relative sugar intake and high-sugar dietary scores did not correlate with the increased risk of AD significantly. In the APOE ε4 status-specific analyses, generally, the effect of sugar intake on all-cause dementia and AD was likely to be more prominent among participants with one APOE ε4 allele. Comprehensive information on the subgroup analyses is summarized in Fig. 4 and Additional file 1: Table S14-S18.

### Mediation analyses

Considering total sugar intake presented a robust significant correlation with dementia in the previous analyses, explorative mediation analyses were conducted to elucidate whether the association between sugar intake and dementia was partly mediated by vascular, inflammatory, and metabolic factors. As shown in Additional file 1: Table S19 and S20, we found that 2.8% and 4.1% of the associations between absolute total sugar intake and all-cause dementia and AD were mediated by SBP, respectively, and 0.7% and 0.4% of the associations between absolute total sugar intake and all-cause dementia and AD were mediated by neutrophil–lymphocyte ratio. In terms of metabolic factors, we did not detect any evidence that metabolic disturbances exerted potential mediation effects in our populations.

### Sensitivity analyses

In the sensitivity analysis excluding participants who had an all-cause dementia event within 3 years of completing their first dietary assessment, the absolute and relative sugar intake and high-sugar dietary scores demonstrated robust significant associations with the risk of all-cause dementia and AD (Additional file 1: Table S21). In the sensitivity analysis which restricted the number of follow-up visits, we first derived three new high-sugar dietary patterns based on populations who completed at least two, three, and four times of dietary assessments, respectively (Additional file 1: Table S22-S24). Most of the subsequent analyses validated the stable significant association between total sugar intake and high-sugar dietary pattern and all-cause dementia, conforming to our primary analyses. However, the statistically significant association between total sugar intake and high-sugar dietary scores and AD incidents only survived when the analytic samples were restricted to participants who completed at least two times of dietary assessments and three times of dietary assessments, respectively. The comprehensive results of sensitivity analyses that restricted the number of follow-up visits are listed in Additional file 1: Table S25-S27.

## Discussion

In the current study, based on a large population-based cohort from UK Biobank, we provided evidence of the longitudinal relationship between sugar intake and high-sugar dietary pattern and dementia. We detected that the increase in daily sugar intake in both absolute and relative forms was significantly associated with the risk of all kinds of dementia, while the high-sugar dietary score was significantly associated with all-cause dementia, but not with AD. The explorative mediation analyses revealed that hypertension, especially high SBP, and systemic inflammation, reflected by neutrophil–lymphocyte ratio, might partly mediate the aforementioned associations to a small degree, providing hints for further mechanisms studies.

To the best of our knowledge, this is the first study to investigate the relationship between high-sugar dietary pattern and dementia in a large-scale and general population. Previous literature regarding high-sugar dietary pattern and dementia is relatively limited, only one study focused on the effect of high-sugar dietary pattern on AD among elderly women in the USA by Liu et al. [17]. They reported that both absolute total sugar intake and a high-sugar diet were associated with the risk of AD significantly. Nonetheless, considering the study population was restricted to elderly women, the generalizability of their conclusion is limited. In the current study, we reconfirmed the significant associations between total sugar intake and high-sugar dietary scores and AD within the female participants. In a general population, we further detected the significant non-linear relationship between sugar intake and AD, whereby higher consumption of more than the median amount of sugar increased the risk of dementia more prominently. Several other studies also investigated the effect of sugar intake on the risk of dementia without consideration of the interaction of different nutrients contained in different food groups in a specific dietary pattern as a whole [16, 31]. Most recently, Schaefer et al. reported that two sugar subtypes (free and intrinsic sugars) from different sources were associated with the risk of dementia [16]. Chong et al. performed the analysis on 1209 participants aged ≥ 60 and found that higher intake of total sugars, free sugar, sucrose, and lactose were significantly associated with lower MMSE scores [31]. Indeed, recent studies focused more on the effect of sugars from sugar-sweetened beverages, an important source of dietary sugar intake, on the risk of dementia [32, 33]. All these investigations together validated the robustness of the finding that sugar intake is tightly correlated with dementia.

The high-sugar dietary pattern established in the present study is characterized by a high-level consumption of fresh and dried fruit, sugar-sweetened beverages, etc. Many fresh fruits are repositories of various bioactive compounds that were reported to play a neuroprotective role [34] and potentially mitigate the adverse impacts of sugar, potentially leading to the underestimation of the association between sugar and AD. As expected, this high-sugar dietary pattern provided us with more comprehensive information, emphasizing the synergistic effects of various foods rather than isolated nutrients. To further clarify, RRR is a data-driven approach to delineate dietary pattern, reflecting combinations of foods consumed by participants with high sugar intake in a large-scale population and could not explain all the variations in the sugar intake. Consequently, not all foods conventionally considered as sugar-rich were positively associated with high-sugar dietary pattern score and there are variances in high-sugar dietary patterns identified by different studies. This requires to be distinguished from hypothesis-driven dietary pattern, such as the Mediterranean diet [35].

Although the *p* values for interactive effects were not stably significant regarding three different exposures and two different outcomes (all-cause dementia and AD), we still conducted subgroup analyses according to age, sex, and APOE status exploratively. Therefore, the results from the subgroup analyses should be integrated cautiously, especially considering the obvious disparities in the number of participants in each group as well as in the statistical power. In the age- and sex-specific analyses, the association between sugar and dementia appeared to be statically significant only in middle-aged females, although the trends were similar in the other subgroups. The exact mechanism is unclear. One possible explanation is that middle-aged females are more vulnerable to systemic inflammation [36] and vascular disorders [37] resulting from excessive sugar intake. In the APOE-specific analyses, the most possible reason for the negative finding in the subgroup with two APOE-ε4 alleles lies in the small sample sizes (less than 100 participants in the analysis of AD). The effect of excessive sugar intake on all-cause dementia and AD was more prominent in APOE-ε4 alleles carriers, potentially partly owing to the complex interplay between sugar intake and metabolism disturbances. Previous studies reported that APOE-ε4 carriers were more likely to develop insulin resistance and exhibited a stronger tendency to develop diabetes [38]. Furthermore, high midlife glycemia was associated with more severe AD neuropathology among APOE-ε4 carriers [39].

In the current study, while the emphasis was placed on the impact of one specific type of carbohydrate, namely sugars, it should be noted that different classes of carbohydrates may share common physiological properties and health effects. Other carbohydrates may also exert significant detrimental effects on dementia. Glycemic load is an important and widely discussed indicator which reflects blood glucose response to a specific ingredient, food, or portion of a meal [19]. A high glycemic load diet always reflects an elevated intake of refined carbohydrates, such as starches and sugars. Gentreau et al. delineated consistent and robust findings that a high glycemic load diet is associated with cognitive decline [40] and an increased risk of dementia [41]. The 2015 Scientific Advisory Committee on Nutrition argued that the cause–effect relationships for outcomes based on variation in diet glycemic load should be integrated with caution, considering the intricate relationship between carbohydrates and glucose [19]. Therefore, we established a high-sugar dietary pattern based on daily sugar intake and food consumption data directly and validated the association between sugar intake and dementia incidents. This body of evidence together suggested that excessive carbohydrate intake (sugars as well as other refined carbohydrates) might have adverse effects on cognitive health, which was of great public health significance.

The underlying mechanisms of such association have not been elucidated clearly so far. Excessive sugar intake might directly cause hyperglycemia and insulin resistance in the brain, disturbing normal brain function and leading to neurodegeneration consequently through a variety of mechanisms, such as glucose neurotoxicity, abnormal energy metabolism, vascular injury, neuroinflammation, and oxidative stress reactions [42–44]. Additionally, it is known that brain-derived neurotrophic factor (BDNF) plays a crucial role in regulating the normal survival and maintenance of neurons and is vital to the process of learning and memory [45]. Previous studies found that a refined sugar diet could reduce the level of hippocampal BDNF, and the function of neuronal plasticity and learning in mouse models [46]. Regarding AD, several studies revealed the interaction between insulin resistance and Aβ accumulation [42, 47], suggesting the role of excessive sugar intake in the neuropathology of AD. Crosstalk between the gut and brain is another alternative hypothesis to link dietary sugar intake with the risk of AD. High-sugar diets have been associated with the disruption of gut microbiota [48], which can contribute to the pathophysiological hallmarks of AD, including oxidative stress, metabolic disturbances, and neuroinflammation through the microbiota–gut–brain axis [49]. Consistent with these proposed potential mechanisms, it might be speculated from our explorative mediation analyses that SBP and neutrophil–lymphocyte ratio, reflecting vascular disorders and systemic inflammation, partly mediated the association between sugar intake and dementia, although the effects were rather moderate (less than 5.0% of the association explained by SBP and less than 1.0% of the association explained by neutrophil–lymphocyte ratio). This indicates that other unmeasured mechanisms of sugar consumption on the risk of dementia are worth further exploration and validation. In the present study, we did not observe any mediating role of BMI and HbA1C, which may be caused by potential reverse causality bias considering the cross-sectional study design of the mediated analyses. That is, participants with diabetes may limit their consumption of sugars and hence demonstrated a negative association between sugar intake and the risk of diabetes, interfering with the interpretation of our findings.

The present study had several other limitations [1]. The food consumption data were collected through a web-based self-reported dietary questionnaire, which might be affected by potential recall bias, resulting in the inaccurate estimation of the consumption of sugar and its subtypes, especially among those participants with prodromal dementia in the current cohort. In addition, diet exposure assessed at baseline might not reflect a long-term habitual dietary pattern and could not capture changes in food preference over time. To be more specific, individuals’ dietary preferences may exhibit variations between weekends and weekdays, as well as across different seasons. However, we performed the sensitivity analyses using diet data from participants who completed at least two, three, and four repeated assessments to better capture the characteristics of their habitual diet [2]. As usually happens with the RRR approach, the identified high-sugar dietary pattern did not explain all variability in the sugar intake, and the remaining variability may result in the underestimation of the effect of sugar intake on dementia [3]. The existence of a “health volunteer” selection bias in the UK Biobank population [50] may contribute to the underestimation of the strength of the positive association. People who actively participate in the UK biobank cohort tend to be healthier than nonparticipants [4]. Most participants in the UK Biobank were white British, which may limit the generalization of our findings [5]. The current study focused on all-cause dementia and its main subtype, Alzheimer’s disease. Future studies are still warranted to clarify the general associations between sugar intake and other types of cognitive decline such as mild cognitive impairment and vascular dementia. The strengths of the study include the large sample size, the prospective study design, and the long-term follow-up. Additionally, the application of RRR to derive the high-sugar dietary pattern has the advantage of addressing the potential interaction of multiple food categories collectively.

One previous report based on the 2009–10 National Health and Nutrition Examination Survey (NHANES) showed that 8.0% and 6.9% of daily energy intake among children/adolescents and adults were from sugar-sweetened beverages in the US [51]. The excess intake of dietary sugar is still a health-threatening issue in both developed and developing countries [7]. Our study confirmed the tight correlation between sugar intake and the risk of dementia, and we first provided evidence that high adherence to the high-sugar dietary pattern was an important modifiable risk factor for dementia, especially all-cause dementia, in a general population. Future studies are warranted to elucidate the underlying mechanisms and provide strong evidence for the causal association between sugar intake and dementia. From a public health perspective, our findings support that controlling the consumption of sugar may be of great significance in preventing the future occurrence of dementia.

## Conclusions

Our study provided evidence that dietary sugar intake was associated with the risk of dementia. Controlling the excess consumption of dietary sugar may be of great public health implications for preventing dementia.

### Supplementary Information

Additional file 1: Table S1-Food groups and their contents. Table S2-UK Biobank codes for dementia diagnosis and classification. Table S3-The variates used in the present study from the UK Biobank. Table S4-Information of missing covariates in model 3. Table S5-Factor loadings for high-sugar dietary pattern among participants completing one dietary assessment. Table S6-Mean sugars intake by quartile of high-sugar dietary pattern score. Table S7-Associations between sugar intake, high-sugar dietary score and all-cause dementia in three models. Table S8-Associations between sugar intake, high-sugar dietary score and Alzheimer’s disease in three models. Table S9-Associations between subtypes of sugars and all-cause dementia in three models. Table S10-Associations between subtypes of sugars and Alzheimer’s disease in three models. Table S11-Inflection points where HR = 1 in the RCS analyses. Table S12-P values on the interaction between exposures and stratification variables with all-cause dementia incidents. Table S13-P values on the interaction between exposures and stratification variables with Alzheimer’s disease incidents. Table S14-Age-specific subgroup analyses of associations between sugar intake, high-sugar dietary score and all-cause dementia. Table S15-Sex-specific subgroup analyses of associations between sugar intake, high-sugar dietary score and all-cause dementia. Table S16-APOE-specific subgroup analyses of associations between sugar intake, high-sugar dietary score and all-cause dementia. Table S17-Age-specific subgroup analyses of associations between sugar intake, high-sugar dietary score and Alzheimer’s disease. Table S18-Sex-specific subgroup analyses of associations between sugar intake, high-sugar dietary score and Alzheimer’s disease. Table S19-Mediation analyses of absolute total sugar intake and dementia. Table S20-Mediation analyses of relative total sugar intake and dementia. Table S21-Sensitivity analysis excluding participants developing all-cause dementia within 3 years. Table S22-Factor loadings for high-sugar dietary pattern among participants who completed at least two dietary assessments. Table S23-Factor loadings for high-sugar dietary pattern among participants who completed at least three dietary assessments. Table S24-Factor loadings for high-sugar dietary pattern among participants who completed at least four dietary assessments. Table S25-Sensitivity analysis among participants who completed at least two dietary assessments. Table S26-Sensitivity analysis among participants who completed at least three dietary assessments. Table S27-Sensitivity analysis among participants who completed at least four dietary assessments.

## Data Availability

The UK Biobank resource is available to researchers who wish to access the data by completing the registration form in the Access Management System (AMS- https://bbams.ndph.ox.ac.uk/ams/).

## Abbreviations

AD: Alzheimer’s disease
BMI: Body mass index
CRP: C-reactive protein
DBP: Diastolic blood pressure
MET: Metabolic equivalent of tasks
RCS: Restricted cubic spline
RRR: Reduced rank regression
SBP: Systolic blood pressure
SSBs: Sugar-sweetened beverages
TDI: Townsend Deprivation Index
