# Supplementary material for: Associations of sugar intake, high-sugar dietary pattern, and the risk of dementia: a prospective cohort study of 210,832 participants
Source: BMC Med. 2024 Jul 18;22:298. doi: 10.1186/s12916-024-03525-6 (PMC11256505; doi:10.1186/s12916-024-03525-6)
Supplement: Supplementary file 1 — Additional file 1: Table S1-Food groups and their contents. Table S2-UK Biobank codes for dementia diagnosis and classification. Table S3-The variates used in the present study from the UK Biobank. Table S4-Information of missing covariates in model 3. Table S5-Factor loadings for high-sugar dietary pattern among participants completing one dietary assessment. Table S6-Mean sugars intake by quartile of high-sugar dietary pattern score. Table S7-Associations between sugar intake, high-sugar dietary score and all-cause dementia in three models. Table S8-Associations between sugar intake, high-sugar dietary score and Alzheimer’s disease in three models. Table S9-Associations between subtypes of sugars and all-cause dementia in three models. Table S10-Associations between subtypes of sugars and Alzheimer’s disease in three models. Table S11-Inflection points where HR = 1 in the RCS analyses. Table S12-P values on the interaction between exposures and stratification variables with all-cause dementia incidents. Table S13-P values on the interaction between exposures and stratification variables with Alzheimer’s disease incidents. Table S14-Age-specific subgroup analyses of associations between sugar intake, high-sugar dietary score and all-cause dementia. Table S15-Sex-specific subgroup analyses of associations between sugar intake, high-sugar dietary score and all-cause dementia. Table S16-APOE-specific subgroup analyses of associations between sugar intake, high-sugar dietary score and all-cause dementia. Table S17-Age-specific subgroup analyses of associations between sugar intake, high-sugar dietary score and Alzheimer’s disease. Table S18-Sex-specific subgroup analyses of associations between sugar intake, high-sugar dietary score and Alzheimer’s disease. Table S19-Mediation analyses of absolute total sugar intake and dementia. Table S20-Mediation analyses of relative total sugar intake and dementia. Table S21-Sensitivity analysis excluding participants developing al [file 12916_2024_3525_MOESM1_ESM.docx]

**Additional file 1**

Table S1-Food groups and their contents

Table S2-UK Biobank codes for dementia diagnosis and classification.

Table S3-The variates used in the present study from the UK Biobank.

Table S4-Information of missing covariates in model 3

Table S5-Factor loadings for high-sugar dietary pattern among participants completing one dietary assessment

Table S6-Mean sugars intake by quartile of high-sugar dietary pattern score

Table S7-Associations between sugar intake, high-sugar dietary score and all-cause dementia in three models

Table S8-Associations between sugar intake, high-sugar dietary score and Alzheimer’s disease in three models

Table S9-Associations between subtypes of sugars and all-cause dementia in three models

Table S10-Associations between subtypes of sugars and Alzheimer’s disease in three models

Table S11-Inflection points where HR=1 in the RCS analyses

Table S12-P values on the interaction between exposures and stratification variables with all-cause dementia incidents

Table S13-P values on the interaction between exposures and stratification variables with Alzheimer’s disease incidents

Table S14-Age-specific subgroup analyses of associations between sugar intake, high-sugar dietary score and all-cause dementia

Table S15-Sex-specific subgroup analyses of associations between sugar intake, high-sugar dietary score and all-cause dementia

Table S16-APOE-specific subgroup analyses of associations between sugar intake, high-sugar dietary score and all-cause dementia

Table S17-Age-specific subgroup analyses of associations between sugar intake, high-sugar dietary score and Alzheimer’s disease

Table S18-Sex-specific subgroup analyses of associations between sugar intake, high-sugar dietary score and Alzheimer’s disease

Table S19-Mediation analyses of absolute total sugar intake and dementia

Table S20-Mediation analyses of relative total sugar intake and dementia

Table S21-Sensitivity analysis excluding participants developing all-cause dementia within 3 years

Table S22-Factor loadings for high-sugar dietary pattern among participants who completed at least two dietary assessments

Table S23-Factor loadings for high-sugar dietary pattern among participants who completed at least three dietary assessments

Table S24-Factor loadings for high-sugar dietary pattern among participants who completed at least four dietary assessments

Table S25-Sensitivity analysis among participants who completed at least two dietary assessments

Table S26-Sensitivity analysis among participants who completed at least three dietary assessments

Table S27-Sensitivity analysis among participants who completed at least four dietary assessments

**Table S1-Food groups and their contents^1^**

| **Food group** | **Food items** |
| --- | --- |
| High-fat milk and cream | Whole milk >3.6 g fat per 100 g |
|  | Cream |
|  | Full fat yogurt |
| Low-fat milk | Semi skimmed milk >1 g fat per 100 g |
|  | Skimmed milk |
|  | Low fat yogurt |
| Non-dairy milk | Rice/oat milk |
|  | Soya |
| High fat cheese | High fat cheese |
| Low fat cheese | Low fat cheese |
| Olive oil | Olive oil (drizzling/dunking) |
| Poultry | Poultry |
| Red meat | Pork |
|  | Beef |
|  | Lamb |
|  | Other meat |
| Processed meat | Processed meat |
| Oily fish | Oily fish |
| Other fish | Shellfish |
|  | White fish & tinned tuna |
| Coated or breaded meat and fish | Breaded/battered chicken |
|  | Breaded/battered fish |
| Egg & egg dishes | Egg & egg dishes |
| Meat substitutes | Meat substitutes - vegetarian |
|  | Meat substitutes - soy |
| Legumes & pulses | Legumes & pulses |
| Vegetables | Raw salad |
|  | Green leafy/cabbages |
|  | Root vegetables |
|  | Tomatoes |
|  | Allium vegetables |
|  | Other vegetables, including mushrooms, fruiting and mixed veg |
|  | Peas/sweetcorn |
| Boiled or baked potatoes | Potatoes/Sweet potatoes (baked/boiled) |
|  | Mashed potatoes |
| Fried or roast potatoes | Fried/roast potatoes |
| Low-fiber bread | White bread |
| High-fiber bread | Wholemeal bread |
|  | Mixed (50/50), brown & seeded |
| Other bread products | Other bread |
|  | Savoury crackers |
|  | Grain dishes - added fat |
|  | Samosa, pakora |
| Fresh fruit | Citrus |
|  | Berries |
|  | Apples & pears |
|  | Other fruit |
| Dried and stewed fruit | Dried fruit |
|  | Stewed fruit |
| Pasta and rice | White pasta & rice |
|  | Sushi |
| Wholemeal pasta, brown rice & other wholegrains | Wholemeal pasta, brown rice & other wholegrains |
| Pizza | Pizza |
| Nuts & seeds | Salted nuts & seeds |
|  | Unsalted nuts & seeds |
| Fruit juice | Fruit juice |
| Coffee and tea | Coffee, caffeinated |
|  | Coffee, decaffeinated |
|  | Tea |
|  | Tea, decaffeinated |
| Sugar-sweetened beverages & other sugary drinks | Sugar-sweetened beverages & other sugary drinks |
| Low/non sugar SSBs | Low/non sugar SSBs |
| Alcoholic drinks(Wine, beer, spirits) | White wine |
|  | Red wine |
|  | Fortified wine |
|  | Beer & Cider |
|  | Spirits |
| Water/Sparkling water | Water/Sparkling water |
| Milk-based and powdered drinks | Milk-based and powdered drinks |
| Soups | Soups |
| Sauces & condiments (high fat) | Sauces & condiments (high fat) |
| Sauces & condiments (low fat) | Sauces & condiments (low fat) |
| Milk-based desserts | Milk-dairy desserts |
|  | Soy desserts and yogurt |
| Grain-based desserts | Other desserts & cakes & pastries |
| Table sugars & preserves | Table sugars & preserves |
| Chocolate and confectionery | Chocolate confectionary |
|  | Other sweets |
| High-fiber breakfast cereals | Bran cereal |
|  | Biscuit cereal |
|  | Porridge |
| Other breakfast cereals | Oat cereal (sugar) |
|  | Muesli |
|  | Other cereal (sugar) |
| Crisps and savoury snacks | Savoury snacks |
| Biscuits | Biscuits |
| Vegetable side dishes and dips | Vegetable side dishes |
|  | Vegetable dips |
| Low animal fat spread | Reduced fat animal fat spread |
| Butter and other animal fat spreads | Butter and other animal fat spreads |
| Lower plant-based fat spread | Reduced fat plant-based fat spread |
| Normal plant-based fat spread | Plant-based fat spread |
| Nut-based spread | Nut-based spread |

# 1.Gao M, Jebb SA, Aveyard P, et al. Associations Between Dietary Patterns and Incident Type 2 Diabetes: Prospective Cohort Study of 120,343 UK Biobank Participants. *Diabetes Care*. Jun 2 2022;45(6):1315-1325. doi:10.2337/dc21-2258

**Table S2-UK Biobank codes for dementia diagnosis and classification**

| **Outcomes** | **International Classification of Diseases-10 (ICD-10)** | **International Classification of Diseases-9 (ICD-9)** |
| --- | --- | --- |
| All-cause dementia | A81.0, F00, F00.0, F00.1, F00.2, F00.9, F01, F01.0, F01.1, F01.2, F01.3, F01.8, F01.9, F02, F02.0, F02.1, F02.2, F02.3, F02.4, F02.8, F03, F05.1, F10.6, G30, G30.0, G30.1, G30.8, G30.9, G31.0, G31.1, G31.8, I67.3 | 290.2, 290.3, 290.4, 291.2, 294.1, 331.0, 331.1, 331.2, 331.5 |
| Alzheimer disease | F00, F00.0, F00.1, F00.2, F00.9, G30, G30.0, G30.1, G30.8, G30.9 | 331.0 |

**Table S3-The variates used in the present study from the UK Biobank.**

| **Variables** | **Field ID** | **Measurements and Assessments** | **Value type** |
| --- | --- | --- | --- |
| **Age** | 21022 | A self-completed touch-screen questionnaire. | Continuous variable |
| **Sex** | 31 | A self-completed touch-screen questionnaire. | Categorical variable |
| **Ethnicity** | 21000 | A self-completed touch-screen questionnaire. | Categorical variable |
| **BMI** | 21001 | BMI is constructed from height and weight measured during the initial Assessment Centre visit. | Continuous variable |
| **Education level** | 6138 | A self-completed touch-screen questionnaire. | Categorical variable |
| **Smoking status** | 20116 | Touchscreen question “Do you smoke tobacco now?” and “In the past, how often have you smoked tobacco?” | Categorical variable |
| **Drinking status** | 20117 | Touchscreen question “About how often do you drink alcohol?” and “Did you previously drink alcohol?” | Categorical variable |
| **Townsend Deprivation Index** | 22189 | Scores were assigned to participants based on their postal codes, with higher scores indicating fewer resources. | Continuous variable |
| **Total MET** | 22040 | International Physical Activity Questionnaire. | Continuous variable |
| **Hypertension** | 6150 | A self-completed touch-screen questionnaire. | Categorical variable |
| **Diabetes** | 2443 | A self-completed touch-screen questionnaire. | Categorical variable |
| **APOE ε4 alleles** | 100315 (Category ID) | Genotype calling was performed by Affymetrix (now part of ThermoFisher Scientific) on two closely related purpose-designed arrays. | Categorical variable |
| **Systolic blood pressure** | 4080, 93 | The method can be viewed using this link **biobank.ndph.ox.ac.uk/ukb/ukb/docs/Bloodpressure.pdf** | Continuous variable |
| **Diastolic blood pressure** | 4079, 94 | The method can be viewed using this link **biobank.ndph.ox.ac.uk/ukb/ukb/docs/Bloodpressure.pdf** | Continuous variable |
| **Neutrophil** | 30140 | The method can be viewed using this link **biobank.ndph.ox.ac.uk/ukb/ukb/docs/haematology.pdf** | Continuous variable |
| **Lymphocyte** | 30120 | The method can be viewed using this link **biobank.ndph.ox.ac.uk/ukb/ukb/docs/haematology.pdf** | Continuous variable |
| **C-reactive protein** | 30710 | The method can be viewed using this link **biobank.ndph.ox.ac.uk/ukb/ukb/docs/biomarker_issues.pdf** | Continuous variable |
| **HbA1c** | 30750 | The method can be viewed using this link **biobank.ndph.ox.ac.uk/ukb/ukb/docs/biomarker_issues.pdf** | Continuous variable |
| **Estimated food nutrients yesterday** | 100117 (Category ID) | Based on the answers to the dietary questionnaire by 24-hour recall through the web-based Oxford WebQ. | Continuous and categorical variables |
| **Dementia** | 42018, 42020, 130836-130843 | Algorithmically-defined outcomes were obtained through algorithmic combinations of coded information from UK Biobank's baseline assessment data collection along with linkage data. The method to identify the first occurrence of health outcomes defined by 3-character ICD10 code can be viewed through this link **biobank.ndph.ox.ac.uk/ukb/ukb/docs/first_occurrences_outcomes.pdf**. | Categorical variable |

# Table S4-Information of missing covariates in model 3^a^

| **Variates** | **No. of sample with missing covariates** |
| --- | --- |
| Ethnicity | 3309 |
| Education | 2660 |
| BMI | 3770 |
| Smoking Status | 3464 |
| Drinking Status | 2894 |
| MET minutes/week | 42159 |
| Townsend Deprivation Index | 2924 |
| Diabetes | 44 |
| Hypertension | 6 |
| *APOE* 4 status | 11574 |
| ^a^Number of sample with missing covariates was calculated based on 210,832 participants. | |

# Table S5-Factor loadings for high-sugar dietary pattern among participants completing one dietary assessment

| Food groups | Factor loadings | Food groups | Factor loadings |
| --- | --- | --- | --- |
| Fresh fruit | 0.599 | Alcoholic drinks(Wine, beer, spirits) | -0.355 |
| Sugar-sweetened beverages & other sugary drinks | 0.354 | Low-fiber bread | -0.263 |
| Fruit juice | 0.318 | High-fiber bread | -0.250 |
| Dried and stewed fruit | 0.282 | Red meat | -0.208 |
| Table sugars & preserves | 0.182 | Pizza | -0.201 |
| Milk-based and powdered drinks | 0.167 | Pasta and rice | -0.198 |
| Chocolate and confectionery | 0.143 | Fried or roast potatoes | -0.170 |
| Vegetables | 0.137 | Other bread products | -0.170 |
| Low-fat milk | 0.126 | Poultry | -0.143 |
| Water/Sparkling water | 0.027 | Egg & egg dishes | -0.135 |
| Milk-based desserts | 0.027 | Crisps and savoury snacks | -0.125 |
| Non-dairy milk | 0.024 | High fat cheese | -0.122 |
| High-fat milk and cream | 0.020 | Processed meat | -0.119 |
| Sauces & condiments (low fat) | 0.018 | Butter and other animal fat spreads | -0.119 |
| High-fiber breakfast cereals | 0.007 | Coated or breaded meat and fish | -0.117 |
|  |  | Nuts & seeds | -0.111 |
|  |  | Boiled or baked potatoes | -0.111 |
|  |  | Oily fish | -0.098 |
|  |  | Wholemeal pasta, brown rice & other wholegrains | -0.073 |
|  |  | Other fish | -0.062 |
|  |  | Biscuits | -0.061 |
|  |  | Normal plant-based fat spread | -0.060 |
|  |  | Meat substitutes | -0.059 |
|  |  | Lower plant-based fat spread | -0.043 |
|  |  | Low animal fat spread | -0.032 |
|  |  | Sauces & condiments (high fat) | -0.027 |
|  |  | Vegetable side dishes and dips | -0.027 |
|  |  | Olive oil | -0.024 |
|  |  | Grain-based desserts | -0.023 |
|  |  | Low fat cheese | -0.018 |
|  |  | Soups | -0.018 |
|  |  | Other breakfast cereals | -0.014 |
|  |  | Nut-based spread | -0.010 |
|  |  | Low/non sugar SSBs | -0.006 |
|  |  | Legumes & pulses | -0.005 |
|  |  | Coffee and tea | -0.001 |

# Table S6-Mean sugars intake by quartile of high-sugar dietary pattern score

| **Means (SD) of sugars intake by high-sugar dietary score** | | | | | | | | |
| --- | --- | --- | --- | --- | --- | --- | --- | --- |
|  | **Q1** | | **Q2** | | **Q3** | | **Q4** | |
| **Type of sugar** | **Mean** | **SD** | **Mean** | **SD** | **Mean** | **SD** | **Mean** | **SD** |
| Absolute fructose sugar intake(g/d) | 18.681 | 10.075 | 23.339 | 10.236 | 29.011 | 10.779 | 41.703 | 16.174 |
| Absolute glucose sugar intake(g/d) | 19.486 | 9.916 | 22.184 | 9.663 | 26.729 | 10.112 | 37.27 | 14.744 |
| Absolute lactose sugar intake(g/d) | 11.995 | 6.873 | 13.381 | 7.004 | 14.455 | 7.504 | 15.951 | 9.025 |
| Absolute sucrose sugar intake(g/d) | 39.38 | 23.354 | 43.116 | 22.289 | 47.913 | 22.777 | 59.734 | 29.01 |
| Absolute maltose sugar intake(g/d) | 11.685 | 10.727 | 6.046 | 4.775 | 4.96 | 3.733 | 4.41 | 3.5 |
| Absolute other sugar intake(g/d) | 1.774 | 2.525 | 2.101 | 2.624 | 2.405 | 2.844 | 3.101 | 3.688 |
| Relative fructose sugar intake(%g/kJ/d) | 0.187 | 0.081 | 0.274 | 0.089 | 0.361 | 0.102 | 0.516 | 0.164 |
| Relative glucose sugar intake(%g/kJ/d) | 0.196 | 0.078 | 0.262 | 0.083 | 0.334 | 0.094 | 0.459 | 0.14 |
| Relative lactose sugar intake(%g/kJ/d) | 0.124 | 0.068 | 0.163 | 0.082 | 0.184 | 0.099 | 0.199 | 0.109 |
| Relative sucrose sugar intake(%g/kJ/d) | 0.39 | 0.177 | 0.502 | 0.19 | 0.588 | 0.212 | 0.718 | 0.249 |
| Relative maltose sugar intake(%g/kJ/d) | 0.117 | 0.1 | 0.071 | 0.051 | 0.06 | 0.04 | 0.052 | 0.034 |
| Relative other sugar intake(%g/kJ/d) | 0.018 | 0.022 | 0.025 | 0.028 | 0.03 | 0.034 | 0.037 | 0.039 |

# Table S7-Associations between sugar intake, high-sugar dietary score and all-cause dementia in three models^a^

|  | **All-cause dementia** | | | | | | | | | | | |
| --- | --- | --- | --- | --- | --- | --- | --- | --- | --- | --- | --- | --- |
|  | **Unadjusted Model 1** | | | | **Adjusted Model 2** | | | | **Adjusted Model 3** | | | |
|  | HR | 95%CI | | P-value | HR | 95%CI | | P-value | HR | 95%CI | | P-value |
| **Absolute sugar intake** | 1.003 | 1.003 | 1.004 | <0.001**^*^** | 1.003 | 1.002 | 1.004 | <0.001**^*^** | 1.003 | 1.002 | 1.004 | 0.001**^*^** |
| **Absolute sugar intake, quartile** |  |  |  |  |  |  |  |  |  |  |  |  |
| Q1(74.134) | Reference | | | | Reference | | | | Reference | | | |
| Q2(105.784)) | 0.871 | 0.759 | 1 | 0.05 | 0.8 | 0.696 | 0.92 | 0.002**^*^** | 0.849 | 0.724 | 0.995 | 0.043**^*^** |
| Q3(133.527) | 1.03 | 0.903 | 1.175 | 0.659 | 0.898 | 0.785 | 1.026 | 0.114 | 0.898 | 0.769 | 1.049 | 0.175 |
| Q4(177.590) | 1.415 | 1.251 | 1.6 | <0.001**^*^** | 1.171 | 1.033 | 1.327 | 0.013**^*^** | 1.171 | 1.013 | 1.355 | 0.033**^*^** |
| **p for trend** | <0.001**^*^** | | | | 0.001**^*^** | | | | 0.003**^*^** | | | |
| **Relative sugar intake** | 1.51 | 1.377 | 1.657 | <0.001**^**^** | 1.347 | 1.218 | 1.488 | <0.001**^*^** | 1.317 | 1.173 | 1.48 | <0.001**^*^** |
| **Relative sugar intake, quartile** |  |  |  |  |  |  |  |  |  |  |  |  |
| Q1(0.980) | Reference | | | | Reference | | | | Reference | | | |
| Q2(1.300) | 1.03 | 0.896 | 1.183 | 0.68 | 0.931 | 0.809 | 1.071 | 0.317 | 0.964 | 0.822 | 1.132 | 0.658 |
| Q3(1.563) | 1.119 | 0.976 | 1.282 | 0.108 | 0.962 | 0.837 | 1.105 | 0.582 | 0.945 | 0.805 | 1.109 | 0.489 |
| Q4(1.957) | 1.613 | 1.423 | 1.83 | <0.001 | 1.331 | 1.17 | 1.513 | <0.001**^*^** | 1.323 | 1.14 | 1.535 | <0.001**^*^** |
| **p for trend** | <0.001**^*^** | | | | <0.001**^*^** | | | | 0.001**^*^** | | | |
| **High-sugar dietary score** | 1.151 | 1.115 | 1.189 | <0.001**^*^** | 1.122 | 1.062 | 1.187 | <0.001**^*^** | 1.09 | 1.045 | 1.136 | 0.001**^*^** |
| **High-sugar dietary score，quartile** |  |  |  |  |  |  |  |  |  |  |  |  |
| Q1(-1.369) | Reference | | | | Reference | | | | Reference | | | |
| Q2(-0.370) | 1.003 | 0.873 | 1.153 | 0.962 | 0.814 | 0.65 | 1.02 | 0.074 | 0.914 | 0.778 | 1.074 | 0.274 |
| Q3(0.348) | 1.161 | 1.015 | 1.328 | 0.030**^*^** | 0.956 | 0.77 | 1.186 | 0.683 | 0.964 | 0.822 | 1.132 | 0.657 |
| **p for trend** | <0.001**^*^** | | | | <0.001**^*^** | | | | 0.001**^*^** | | | |

**^a Model 1 was unadjusted. Model 2 was adjusted for baseline demographic information and socio-economic factors including sex, age at baseline, ethnicity, education level, BMI and TDI. Model 3 was further adjusted for important lifestyle factors, comorbidities and genotype factor including smoking status, drinking status, metabolic equivalent of tasks (MET), diabetes, hypertension, and Apolipoprotein E (APOE) ε4 status. *Statistically significant difference was found among the groups (p<0.05).Abbreviation: HR, Hazard ratios; 95%CI, Confidential intervals^**

# Table S8-Associations between sugar intake, high-sugar dietary score and Alzheimer’s disease in three models^a^

|  | **Alzheimer's disease** | | | | | | | | | | | |
| --- | --- | --- | --- | --- | --- | --- | --- | --- | --- | --- | --- | --- |
|  | **Unadjusted Model 1** | | | | **Adjusted Model 2** | | | | **Adjusted Model 3** | | | |
|  | **HR** | **95%CI** | | **P-value** | **HR** | **95%CI** | | **P-value** | **HR** | **95%CI** | | **P-value** |
| **Absolute sugar intake** | 1.004 | 1.003 | 1.005 | <0.001^*^ | 1.003 | 1.002 | 1.004 | <0.001^*^ | 1.002 | 1.001 | 1.004 | 0.005^*^ |
| **Absolute sugar intake, quartile** |  | | | |  | | | |  | | | |
| Q1(74.134) | Reference | | | | Reference | | | | Reference | | | |
| Q2(105.784)) | 0.919 | 0.738 | 1.145 | 0.452 | 0.843 | 0.675 | 1.052 | 0.131 | 0.914 | 0.712 | 1.174 | 0.483 |
| Q3(133.527) | 1.215 | 0.989 | 1.491 | 0.063 | 1.073 | 0.872 | 1.32 | 0.507 | 0.993 | 0.779 | 1.267 | 0.958 |
| Q4(177.590) | 1.572 | 1.294 | 1.91 | <0.001^*^ | 1.295 | 1.063 | 1.579 | 0.010^*^ | 1.191 | 0.944 | 1.503 | 0.14 |
| **p for trend** | <0.001^*^ | | | | <0.001^*^ | | | | 0.058 | | | |
| **Relative sugar intake** | 1.634 | 1.42 | 1.881 | <0.001^*^ | 1.4 | 1.201 | 1.631 | <0.001^*^ | 1.249 | 1.041 | 1.5 | 0.017^*^ |
| **Relative sugar intake, quartile** |  | | | |  | | | |  | | | |
| Q1(0.980) | Reference | | | | Reference | | | | Reference | | | |
| Q2(1.300) | 1.002 | 0.804 | 1.248 | 0.989 | 0.884 | 0.708 | 1.104 | 0.277 | 0.983 | 0.764 | 1.265 | 0.892 |
| Q3(1.563) | 1.136 | 0.917 | 1.409 | 0.243 | 0.947 | 0.763 | 1.176 | 0.622 | 0.938 | 0.729 | 1.207 | 0.619 |
| Q4(1.957) | 1.781 | 1.466 | 2.164 | <0.001^*^ | 1.396 | 1.145 | 1.702 | 0.001^*^ | 1.283 | 1.014 | 1.624 | 0.038^*^ |
| **p for trend** | <0.001^*^ | | | | <0.001^*^ | | | | 0.021^*^ | | | |
| **High-sugar dietary score** | 1.191 | 1.136 | 1.248 | <0.001^*^ | 1.122 | 1.062 | 1.187 | <0.001^*^ | 1.067 | 0.998 | 1.14 | 0.056 |
| **High-sugar dietary score, quartile** |  | | | |  | | | |  | | | |
| Q1(-1.369) | Reference | | | | Reference | | | | Reference | | | |
| Q2(-0.370) | 0.94 | 0.753 | 1.173 | 0.582 | 0.814 | 0.65 | 1.02 | 0.074 | 0.871 | 0.674 | 1.125 | 0.29 |
| Q3(0.348) | 1.16 | 0.94 | 1.432 | 0.166 | 0.956 | 0.77 | 1.186 | 0.683 | 0.97 | 0.756 | 1.246 | 0.813 |
| Q4(1.364) | 1.737 | 1.431 | 2.109 | <0.001^*^ | 1.349 | 1.102 | 1.652 | 0.004^*^ | 1.196 | 0.942 | 1.52 | 0.142 |
| **p for trend** | <0.001^*^ | | | | <0.001^*^ | | | | 0.053 | | | |

**^a Model 1 was unadjusted. Model 2 was adjusted for baseline demographic information and socio-economic factors including sex, age at baseline, ethnicity, education level, BMI and TDI. Model 3 was further adjusted for important lifestyle factors, comorbidities and genotype factor including smoking status, drinking status, metabolic equivalent of tasks (MET), diabetes, hypertension, and Apolipoprotein E (APOE) ε4 status. *Statistically significant difference was found among the groups (p<0.05). Abbreviation: HR, Hazard ratios; 95%CI, Confidential intervals^**

# Table S9-Associations between subtypes of sugars and all-cause dementia in three models^a^

|  | **All-cause dementia** | | | | | | | | | | | |
| --- | --- | --- | --- | --- | --- | --- | --- | --- | --- | --- | --- | --- |
|  | **Unadjusted Model 1** | | | | **Adjusted Model 2** | | | | **Adjusted Model 3** | | | |
|  | **HR** | **95%CI** | | **P-value** | **HR** | **95%CI** | | **P-value** | **HR** | **95%CI** | | **P-value** |
| Total fructose intake | 1.01 | 1.007 | 1.012 | 0.006^*^ | 1.006 | 1.003 | 1.009 | <0.001^*^ | 1.005 | 1.002 | 1.009 | 0.012^*^ |
| Total fructose intake, quartile |  | | | |  | | | |  | | | |
| Q1(12.891) | Reference | | | | Reference | | | | Reference | | | |
| Q2(22.185) | 0.945 | 0.826 | 1.082 | 0.413 | 0.848 | 0.74 | 0.973 | 0.108 | 0.858 | 0.734 | 1.003 | 0.055 |
| Q3(30.593) | 1.066 | 0.935 | 1.215 | 0.338 | 0.908 | 0.795 | 1.037 | 0.155 | 0.871 | 0.746 | 1.017 | 0.08 |
| Q4(44.017) | 1.317 | 1.162 | 1.492 | <0.001^*^ | 1.065 | 0.937 | 1.209 | 0.336 | 1.045 | 0.903 | 1.21 | 0.555 |
| p for trend | <0.001^*^ | | | | 0.27 | | | | 0.159 | | | |
| Total glucose intake | 1.011 | 1.008 | 1.014 | 0.006^*^ | 1.007 | 1.004 | 1.01 | <0.001^*^ | 1.006 | 1.002 | 1.009 | 0.004^*^ |
| Total glucose intake, quartile |  | | | |  | | | |  | | | |
| Q1(12.997) | Reference | | | | Reference | | | | Reference | | | |
| Q2(21.148) | 0.84 | 0.732 | 0.963 | 0.072 | 0.767 | 0.668 | 0.881 | <0.001^*^ | 0.776 | 0.662 | 0.909 | 0.002^*^ |
| Q3(28.436) | 1.077 | 0.948 | 1.224 | 0.256 | 0.941 | 0.826 | 1.071 | 0.357 | 0.931 | 0.801 | 1.081 | 0.349 |
| Q4(40.339) | 1.245 | 1.1 | 1.409 | 0.006^*^ | 1.008 | 0.888 | 1.143 | 0.903 | 0.993 | 0.859 | 1.149 | 0.927 |
| p for trend | <0.001^*^ | | | | 0.165 | | | | 0.347 | | | |
| Total lactose intake | 1.014 | 1.009 | 1.02 | <0.001^*^ | 1.006 | 1 | 1.012 | 0.216 | 1.005 | 0.998 | 1.012 | 0.138 |
| Total lactose intake, quartile |  | | | |  | | | |  | | | |
| Q1(5.509) | Reference | | | | Reference | | | | Reference | | | |
| Q2(11.023) | 0.981 | 0.858 | 1.121 | 0.776 | 0.903 | 0.789 | 1.034 | 0.142 | 0.909 | 0.779 | 1.062 | 0.23 |
| Q3(15.451) | 1.095 | 0.961 | 1.247 | 0.173 | 0.938 | 0.822 | 1.071 | 0.346 | 0.952 | 0.819 | 1.106 | 0.518 |
| Q4(22.269) | 1.275 | 1.124 | 1.446 | 0.006^*^ | 1.041 | 0.916 | 1.182 | 0.54 | 1.003 | 0.866 | 1.161 | 0.973 |
| p for trend | <0.001^*^ | | | | 0.316 | | | | 0.740 | | | |
| Total sucrose intake | 1.006 | 1.004 | 1.007 | <0.001^*^ | 1.005 | 1.004 | 1.007 | <0.001^*^ | 1.006 | 1.004 | 1.008 | <0.001^*^ |
| Total sucrose intake, quartile |  | | | |  | | | |  | | | |
| Q1(22.855) | Reference | | | | Reference | | | | Reference | | | |
| Q2(36.641) | 0.866 | 0.754 | 0.995 | 0.258 | 0.831 | 0.722 | 0.957 | 0.06 | 0.792 | 0.673 | 0.932 | 0.005^*^ |
| Q3(49.927) | 1.16 | 1.02 | 1.321 | 0.144 | 1.086 | 0.953 | 1.237 | 0.218 | 1.103 | 0.951 | 1.281 | 0.196 |
| Q4(73.958) | 1.366 | 1.206 | 1.548 | <0.001^*^ | 1.253 | 1.104 | 1.423 | <0.001^*^ | 1.253 | 1.083 | 1.45 | 0.002^*^ |
| p for trend | <0.001^*^ | | | | <0.001^*^ | | | | <0.001^*^ | | | |
| Total maltose intake | 1 | 0.993 | 1.006 | 0.912 | 1.001 | 0.994 | 1.008 | 0.81 | 1.001 | 0.993 | 1.01 | 0.779 |
| Total maltose intake, quartile |  | | | |  | | | |  | | | |
| Q1(0.029) | Reference | | | | Reference | | | | Reference | | | |
| Q2(0.050) | 1.066 | 0.936 | 1.214 | 0.335 | 1.021 | 0.895 | 1.165 | 0.755 | 1.002 | 0.861 | 1.166 | 0.983 |
| Q3(0.069) | 1.078 | 0.947 | 1.227 | 0.254 | 1.03 | 0.901 | 1.177 | 0.664 | 1.047 | 0.899 | 1.219 | 0.557 |
| Q4(0.118) | 1.115 | 0.98 | 1.268 | 0.099 | 1.086 | 0.944 | 1.25 | 0.247 | 1.05 | 0.893 | 1.234 | 0.554 |
| p for trend | 0.137 | | | | 0.229 | | | | 0.517 | | | |
| Total other sugar intake | 1.024 | 1.011 | 1.036 | <0.001^*^ | 1.025 | 1.012 | 1.038 | <0.001^*^ | 1.023 | 1.008 | 1.039 | 0.003^*^ |
| Total other sugars intake, quartile |  | | | |  | | | |  | | | |
| Q1(0.243) | Reference | | | | Reference | | | | Reference | | | |
| Q2(1.275) | 0.909 | 0.798 | 1.036 | 0.153 | 0.899 | 0.788 | 1.027 | 0.116 | 0.84 | 0.722 | 0.977 | 0.024^*^ |
| Q3(2.177) | 0.951 | 0.836 | 1.082 | 0.443 | 0.93 | 0.816 | 1.061 | 0.28 | 0.871 | 0.75 | 1.012 | 0.072 |
| Q4(4.232) | 1.112 | 0.982 | 1.259 | 0.094 | 1.089 | 0.96 | 1.236 | 0.185 | 1.023 | 0.885 | 1.182 | 0.759 |
| p for trend | 0.138 | | | | 0.053 | | | | 0.331 | | | |
| Relative fructose intake | 2.03 | 1.577 | 2.612 | <0.001^*^ | 1.48 | 1.126 | 1.946 | 0.06 | 1.372 | 1 | 1.883 | 0.05 |
| Relative fructose intake, quartile |  | | | |  | | | |  | | | |
| Q1(0.160) | Reference | | | | Reference | | | | Reference | | | |
| Q2(0.266) | 0.986 | 0.861 | 1.13 | 0.842 | 0.872 | 0.76 | 1.001 | 0.052 | 0.914 | 0.781 | 1.069 | 0.26 |
| Q3(0.363) | 1.061 | 0.932 | 1.207 | 0.37 | 0.892 | 0.781 | 1.018 | 0.089 | 0.855 | 0.733 | 0.997 | 0.27 |
| Q4(0.523) | 1.328 | 1.171 | 1.506 | <0.001^*^ | 1.097 | 0.964 | 1.249 | 0.161 | 1.091 | 0.939 | 1.266 | 0.256 |
| p for trend | <0.001^*^ | | | | 0.045 | | | | 0.159 | | | |
| Relative glucose intake | 2.324 | 1.729 | 3.125 | <0.001^*^ | 1.539 | 1.12 | 2.117 | 0.048^*^ | 1.375 | 0.95 | 1.99 | 0.092 |
| Relative glucose intake, quartile |  | | | |  | | | |  | | | |
| Q1(0.169) | Reference | | | | Reference | | | | Reference | | | |
| Q2(0.265) | 0.955 | 0.839 | 1.086 | 0.48 | 0.853 | 0.748 | 0.972 | 0.102 | 0.839 | 0.721 | 0.975 | 0.132 |
| Q3(0.346) | 1.078 | 0.941 | 1.236 | 0.277 | 0.913 | 0.795 | 1.049 | 0.198 | 0.905 | 0.772 | 1.061 | 0.22 |
| Q4(0.469) | 1.331 | 1.176 | 1.505 | <0.001^*^ | 1.106 | 0.975 | 1.256 | 0.118 | 1.078 | 0.933 | 1.247 | 0.309 |
| p for trend | <0.001^*^ | | | | 0.120 | | | | 0.089 | | | |
| Relative lactose intake | 2.47 | 1.627 | 3.751 | <0.001^*^ | 1.439 | 0.897 | 2.31 | 0.131 | 1.24 | 0.714 | 2.154 | 0.444 |
| Relative lactose intake, quartile |  | | | |  | | | |  | | | |
| Q1(0.065) | Reference | | | | Reference | | | | Reference | | | |
| Q2(0.131) | 0.964 | 0.845 | 1.1 | 0.586 | 0.874 | 0.765 | 0.998 | 0.282 | 0.899 | 0.772 | 1.047 | 0.172 |
| Q3(0.187) | 1.012 | 0.885 | 1.158 | 0.863 | 0.872 | 0.761 | 1 | 0.05 | 0.879 | 0.752 | 1.029 | 0.108 |
| Q4(0.271) | 1.295 | 1.141 | 1.471 | <0.001^*^ | 1.068 | 0.938 | 1.216 | 0.322 | 1.062 | 0.914 | 1.233 | 0.432 |
| p for trend | <0.001^*^ | | | | 0.139 | | | | 0.278 | | | |
| Relative sucrose intake | 1.797 | 1.535 | 2.103 | <0.001^*^ | 1.746 | 1.471 | 2.072 | <0.001^*^ | 1.772 | 1.458 | 2.154 | <0.001^*^ |
| Relative sucrose intake, quartile |  | | | |  | | | |  | | | |
| Q1(0.305) | Reference | | | | Reference | | | | Reference | | | |
| Q2(0.456) | 1.05 | 0.916 | 1.204 | 0.481 | 1.003 | 0.874 | 1.152 | 0.964 | 0.992 | 0.847 | 1.163 | 0.924 |
| Q3(0.587) | 1.181 | 1.033 | 1.349 | 0.084 | 1.098 | 0.96 | 1.256 | 0.173 | 1.072 | 0.918 | 1.252 | 0.378 |
| Q4(0.802) | 1.447 | 1.275 | 1.643 | <0.001^*^ | 1.345 | 1.182 | 1.53 | <0.001^*^ | 1.353 | 1.166 | 1.569 | <0.001^*^ |
| p for trend | <0.001^*^ | | | | <0.001^*^ | | | | <0.001^*^ | | | |
| Relative maltose intake | 1.07 | 0.545 | 2.1 | 0.845 | 1.193 | 0.572 | 2.488 | 0.638 | 1.237 | 0.534 | 2.865 | 0.619 |
| Relative maltose intake, quartile |  | | | |  | | | |  | | | |
| Q1(0.029) | Reference | | | | Reference | | | | Reference | | | |
| Q2(0.050) | 1.03 | 0.91 | 1.166 | 0.638 | 0.997 | 0.879 | 1.13 | 0.96 | 0.962 | 0.832 | 1.111 | 0.597 |
| Q3(0.069) | 1.032 | 0.898 | 1.186 | 0.655 | 0.994 | 0.863 | 1.145 | 0.934 | 0.991 | 0.844 | 1.165 | 0.914 |
| Q4(0.118) | 1.069 | 0.946 | 1.208 | 0.283 | 1.064 | 0.933 | 1.213 | 0.353 | 1.02 | 0.878 | 1.185 | 0.799 |
| p for trend | 0.293 | | | | 0.290 | | | | 0.644 | | | |
| Relative other sugar intake | 3.53 | 1.001 | 12.444 | 0.05 | 5.727 | 1.529 | 21.458 | 0.06 | 4.427 | 0.91 | 21.536 | 0.065 |
| Relative other sugars intake, quartile |  | | | |  | | | |  | | | |
| Q1(0.003) | Reference | | | | Reference | | | | Reference | | | |
| Q2(0.015) | 0.933 | 0.818 | 1.065 | 0.307 | 0.919 | 0.804 | 1.051 | 0.217 | 0.854 | 0.733 | 0.995 | 0.284 |
| Q3(0.025) | 0.972 | 0.846 | 1.116 | 0.685 | 0.943 | 0.819 | 1.086 | 0.415 | 0.89 | 0.758 | 1.046 | 0.158 |
| Q4(0.045) | 1.131 | 1.006 | 1.272 | 0.040^*^ | 1.138 | 1.009 | 1.284 | 0.036^*^ | 1.06 | 0.924 | 1.217 | 0.405 |
| p for trend | 0.054 | | | | 0.036^*^ | | | | 0.126 | | | |

**^a^ Model 1 was unadjusted.**

**Model 2 was adjusted for baseline demographic information and socio-economic factors including sex, age at baseline, ethnicity, education level, BMI and TDI.**

**Model 3 was further adjusted for important lifestyle factors, comorbidities and genotype factor including smoking status, drinking status, metabolic equivalent of tasks (MET), diabetes, hypertension, and Apolipoprotein E (APOE) ε4 status.**

**^*^Statistically significant difference was found among the groups after Bonferroni correction (p<0.05).**

**Abbreviation: HR, Hazard ratios; 95%CI, Confidential intervals**

# Table S10-Associations between subtypes of sugars and Alzheimer’s disease in three models^a^

|  | **Alzheimer's disease** | | | | | | | | | | | |
| --- | --- | --- | --- | --- | --- | --- | --- | --- | --- | --- | --- | --- |
|  | **Unadjusted Model 1** | | | | **Adjusted Model 2** | | | | **Adjusted Model 3** | | | |
|  | **HR** | **95%CI** | | **P-value** | **HR** | **95%CI** | | **P-value** | **HR** | **95%CI** | | **P-value** |
| Total fructose intake | 1.013 | 1.009 | 1.016 | <0.001^*^ | 1.009 | 1.005 | 1.014 | <0.001^*^ | 1.006 | 1 | 1.011 | 0.22 |
| Total fructose intaker,quartile |  | | | |  | | | |  | | | |
| Q1(12.891) | Reference | | | | Reference | | | | Reference | | | |
| Q2(22.185) | 1.016 | 0.817 | 1.265 | 0.884 | 0.926 | 0.742 | 1.155 | 0.495 | 0.886 | 0.689 | 1.14 | 0.348 |
| Q3(30.593) | 1.297 | 1.055 | 1.595 | 0.081 | 1.108 | 0.898 | 1.367 | 0.339 | 1.06 | 0.834 | 1.348 | 0.633 |
| Q4(44.017) | 1.587 | 1.302 | 1.936 | <0.001^*^ | 1.271 | 1.038 | 1.556 | 0.121 | 1.085 | 0.858 | 1.372 | 0.497 |
| p for trend | <0.001^*^ | | | | 0.017^*^ | | | | 0.308 | | | |
| Total glucose intake | 1.014 | 1.006 | 1.023 | 0.001^*^ | 1.01 | 1.005 | 1.015 | <0.001^*^ | 1.006 | 1.001 | 1.012 | 0.181 |
| Total glucose intake,quartile |  | | | |  | | | |  | | | |
| Q1(12.997) | Reference | | | | Reference | | | | Reference | | | |
| Q2(21.148) | 0.893 | 0.717 | 1.111 | 0.31 | 0.829 | 0.665 | 1.034 | 0.096 | 0.857 | 0.665 | 1.103 | 0.231 |
| Q3(28.436) | 1.211 | 0.988 | 1.483 | 0.065 | 1.059 | 0.862 | 1.301 | 0.584 | 1.07 | 0.843 | 1.357 | 0.579 |
| Q4(40.339) | 1.488 | 1.225 | 1.807 | <0.001^*^ | 1.199 | 0.984 | 1.461 | 0.072 | 1.085 | 0.86 | 1.369 | 0.492 |
| p for trend | <0.001^*^ | | | | 0.007^*^ | | | | 0.197 | | | |
| Total lactose intake | 1.014 | 1.006 | 1.023 | 0.001^*^ | 1.005 | 0.997 | 1.014 | 0.228 | 1 | 0.989 | 1.011 | 0.991 |
| Total lactose intake, quartile |  | | | |  | | | |  | | | |
| Q1(5.509) | Reference | | | | Reference | | | | Reference | | | |
| Q2(11.023) | 1.082 | 0.875 | 1.338 | 0.466 | 1.014 | 0.818 | 1.256 | 0.9 | 0.96 | 0.751 | 1.227 | 0.743 |
| Q3(15.451) | 1.348 | 1.101 | 1.649 | 0.023^*^ | 1.162 | 0.947 | 1.426 | 0.149 | 1.157 | 0.92 | 1.456 | 0.213 |
| Q4(22.269) | 1.326 | 1.083 | 1.624 | 0.038^*^ | 1.08 | 0.88 | 1.326 | 0.46 | 0.912 | 0.718 | 1.158 | 0.449 |
| p for trend | 0.002^*^ | | | | 0.329 | | | | 0.657 | | | |
| Total sucrose intake | 1.006 | 1.003 | 1.008 | <0.001^*^ | 1.005 | 1.002 | 1.007 | <0.001^*^ | 1.004 | 1.001 | 1.007 | 0.051 |
| Total sucrose intake,quartile |  | | | |  | | | |  | | | |
| Q1(22.855) | Reference | | | | Reference | | | | Reference | | | |
| Q2(36.641) | 0.934 | 0.752 | 1.16 | 0.537 | 0.904 | 0.726 | 1.124 | 0.363 | 0.839 | 0.652 | 1.08 | 0.173 |
| Q3(49.927) | 1.3 | 1.064 | 1.589 | 0.062 | 1.222 | 0.998 | 1.497 | 0.052 | 1.153 | 0.915 | 1.454 | 0.228 |
| Q4(73.958) | 1.393 | 1.143 | 1.697 | 0.006^*^ | 1.275 | 1.042 | 1.559 | 0.108 | 1.164 | 0.923 | 1.469 | 0.2 |
| p for trend | <0.001^*^ | | | | 0.002^*^ | | | | 0.042^*^ | | | |
| Total maltose intake | 0.998 | 0.988 | 1.008 | 0.684 | 1.003 | 0.992 | 1.014 | 0.616 | 1.006 | 0.993 | 1.019 | 0.391 |
| Total maltose intake,quartile |  | | | |  | | | |  | | | |
| Q1(0.029) | Reference | | | | Reference | | | | Reference | | | |
| Q2(0.050) | 0.963 | 0.792 | 1.172 | 0.709 | 0.948 | 0.778 | 1.155 | 0.596 | 0.929 | 0.738 | 1.171 | 0.534 |
| Q3(0.069) | 0.897 | 0.735 | 1.095 | 0.284 | 0.873 | 0.711 | 1.072 | 0.194 | 0.879 | 0.693 | 1.115 | 0.289 |
| Q4(0.118) | 0.962 | 0.79 | 1.17 | 0.696 | 0.989 | 0.798 | 1.225 | 0.919 | 0.993 | 0.775 | 1.272 | 0.955 |
| p for trend | 0.773 | | | | 0.916 | | | | 0.849 | | | |
| Total other sugar intake | 1.023 | 1.003 | 1.042 | 0.131 | 1.021 | 1.001 | 1.042 | 0.247 | 1.023 | 0.999 | 1.048 | 0.063 |
| Total other sugars intake, quartile |  | | | |  | | | |  | | | |
| Q1(0.243) | Reference | | | | Reference | | | | Reference | | | |
| Q2(1.275) | 0.87 | 0.709 | 1.067 | 0.181 | 0.842 | 0.685 | 1.034 | 0.101 | 0.784 | 0.62 | 0.991 | 0.25 |
| Q3(2.177) | 0.93 | 0.761 | 1.136 | 0.479 | 0.881 | 0.719 | 1.08 | 0.222 | 0.759 | 0.599 | 0.961 | 0.131 |
| Q4(4.232) | 1.134 | 0.937 | 1.373 | 0.195 | 1.081 | 0.891 | 1.311 | 0.432 | 0.966 | 0.774 | 1.206 | 0.758 |
| p for trend | 0.006^*^ | | | | 0.171 | | | | 0.843 | | | |
| Relative fructose intake | 3.04 | 2.098 | 4.405 | <0.001^*^ | 2.041 | 1.358 | 3.069 | 0.001^*^ | 1.511 | 0.93 | 2.457 | 0.096 |
| Relative fructose intake, quartile |  | | | |  | | | |  | | | |
| Q1(0.160) | Reference | | | | Reference | | | | Reference | | | |
| Q2(0.266) | 1.165 | 0.94 | 1.444 | 0.164 | 1.018 | 0.819 | 1.266 | 0.874 | 1.142 | 0.89 | 1.465 | 0.297 |
| Q3(0.363) | 1.216 | 0.988 | 1.495 | 0.065 | 1.018 | 0.825 | 1.257 | 0.867 | 1.028 | 0.803 | 1.316 | 0.826 |
| Q4(0.523) | 1.592 | 1.304 | 1.945 | <0.001^*^ | 1.259 | 1.024 | 1.547 | 0.173 | 1.172 | 0.918 | 1.496 | 0.203 |
| p for trend | <0.001^*^ | | | | 0.017^*^ | | | | 0.308 | | | |
| Relative glucose intake | 3.584 | 2.324 | 5.526 | <0.001^*^ | 2.174 | 1.352 | 3.497 | 0.008^*^ | 1.6 | 0.908 | 2.817 | 0.104 |
| Relative glucose intake, quartile |  | | | |  | | | |  | | | |
| Q1(0.169) | Reference | | | | Reference | | | | Reference | | | |
| Q2(0.265) | 0.98 | 0.798 | 1.204 | 0.848 | 0.881 | 0.716 | 1.084 | 0.231 | 1.009 | 0.794 | 1.283 | 0.943 |
| Q3(0.346) | 1.095 | 0.882 | 1.361 | 0.41 | 0.919 | 0.737 | 1.145 | 0.451 | 0.96 | 0.741 | 1.243 | 0.757 |
| Q4(0.469) | 1.585 | 1.31 | 1.918 | 0.000^*^ | 1.271 | 1.045 | 1.546 | 0.098 | 1.245 | 0.987 | 1.569 | 0.064 |
| p for trend | <0.001^*^ | | | | 0.003^*^ | | | | 0.047^*^ | | | |
| Relative lactose intake | 2.433 | 1.27 | 4.662 | 0.044^*^ | 1.258 | 0.603 | 2.628 | 0.541 | 0.913 | 0.381 | 2.188 | 0.838 |
| Relative lactose intake, quartile |  | | | |  | | | |  | | | |
| Q1(0.065) | Reference | | | | Reference | | | | Reference | | | |
| Q2(0.131) | 1.055 | 0.86 | 1.294 | 0.609 | 0.959 | 0.78 | 1.179 | 0.691 | 0.96 | 0.76 | 1.213 | 0.732 |
| Q3(0.187) | 1.037 | 0.839 | 1.283 | 0.736 | 0.882 | 0.711 | 1.093 | 0.252 | 0.791 | 0.616 | 1.016 | 0.067 |
| Q4(0.271) | 1.381 | 1.131 | 1.686 | 0.009^*^ | 1.104 | 0.902 | 1.353 | 0.337 | 1.021 | 0.808 | 1.29 | 0.863 |
| p for trend | 0.001^*^ | | | | 0.309 | | | | 0.997 | | | |
| Relative sucrose intake | 1.739 | 1.357 | 2.229 | <0.001^*^ | 1.607 | 1.222 | 2.113 | 0.001^*^ | 1.426 | 1.024 | 1.985 | 0.216 |
| Relative sucrose intake, quartile |  | | | |  | | | |  | | | |
| Q1(0.305) | Reference | | | | Reference | | | | Reference | | | |
| Q2(0.456) | 1.155 | 0.935 | 1.427 | 0.181 | 1.08 | 0.873 | 1.336 | 0.479 | 1.03 | 0.806 | 1.315 | 0.815 |
| Q3(0.587) | 1.219 | 0.989 | 1.502 | 0.064 | 1.092 | 0.884 | 1.347 | 0.414 | 1.034 | 0.811 | 1.317 | 0.789 |
| Q4(0.802) | 1.509 | 1.237 | 1.842 | <0.001^*^ | 1.344 | 1.098 | 1.643 | 0.024^*^ | 1.23 | 0.973 | 1.554 | 0.084 |
| p for trend | <0.001^*^ | | | | 0.003^*^ | | | | 0.069 | | | |
| Relative maltose intake | 0.69 | 0.231 | 2.062 | 0.506 | 1.034 | 0.319 | 3.353 | 0.956 | 1.666 | 0.452 | 6.139 | 0.443 |
| Relative maltose intake, quartile |  | | | |  | | | |  | | | |
| Q1(0.029) | Reference | | | | Reference | | | | Reference | | | |
| Q2(0.050) | 0.954 | 0.791 | 1.151 | 0.624 | 0.941 | 0.778 | 1.138 | 0.529 | 0.912 | 0.729 | 1.14 | 0.418 |
| Q3(0.069) | 0.885 | 0.713 | 1.099 | 0.269 | 0.866 | 0.695 | 1.08 | 0.201 | 0.884 | 0.686 | 1.14 | 0.343 |
| Q4(0.118) | 0.936 | 0.776 | 1.129 | 0.488 | 0.974 | 0.797 | 1.191 | 0.798 | 1 | 0.793 | 1.26 | 0.998 |
| p for trend | 0.519 | | | | 0.894 | | | | 0.822 | | | |
| Relative other sugar intake | 2.137 | 0.274 | 16.659 | 0.469 | 2.28 | 0.253 | 20.575 | 0.463 | 2.306 | 0.172 | 30.988 | 0.528 |
| Relative other sugars intake, quartile |  | | | |  | | | |  | | | |
| Q1(0.003) | Reference | | | | Reference | | | | Reference | | | |
| Q2(0.015) | 0.887 | 0.721 | 1.092 | 0.26 | 0.855 | 0.693 | 1.054 | 0.141 | 0.767 | 0.602 | 0.977 | 0.189 |
| Q3(0.025) | 1.011 | 0.817 | 1.25 | 0.922 | 0.947 | 0.764 | 1.175 | 0.623 | 0.87 | 0.679 | 1.115 | 0.272 |
| Q4(0.045) | 1.138 | 0.949 | 1.365 | 0.162 | 1.096 | 0.91 | 1.32 | 0.334 | 0.972 | 0.785 | 1.203 | 0.792 |
| p for trend | <0.001^*^ | | | | 0.119 | | | | 0.715 | | | |

**^a^ Model 1 was unadjusted.**

**Model 2 was adjusted for baseline demographic information and socio-economic factors including sex, age at baseline, ethnicity, education level, BMI and TDI.**

**Model 3 was further adjusted for important lifestyle factors, comorbidities and genotype factor including smoking status, drinking status, metabolic equivalent of tasks (MET), diabetes, hypertension, and Apolipoprotein E (APOE) ε4 status.**

**^*^Statistically significant difference was found among the groups after Bonferroni correction (p<0.05).**

**Abbreviation: HR, Hazard ratios; 95%CI, Confidential intervals**

# Table S11-Inflection points where HR=1 in the RCS analyses

|  | All-cause dementia | | Alzheimer's dementia | |
| --- | --- | --- | --- | --- |
|  | Point 1 | Point 2(median) | Point 1 | Point 2(median) |
| Absolute sugar intake(g/d) | 99 | 119.183 | 93.42 | 119.183 |
| Relative sugar intake(%g/kJ/d) | 1.13 | 1.429 | 1.105 | 1.429 |
| High-sugar dietary score | -0.906 | -0.013 | -1.001 | -0.013 |

# Table S12-P values on the interaction between exposures and stratification variables with all-cause dementia incidents^a^

|  | **All-cause dementia** | | | | | |
| --- | --- | --- | --- | --- | --- | --- |
|  | Age | Sex | BMI | APOE state | Diabetes | Hypertension |
| Absolute sugar intake | 0.373 | 0.013^*^ | 0.438 | 0.233 | 0.527 | 0.005^*^ |
| Absolute sugar intake, quartile |  |  |  |  |  |  |
| Q1 | Reference | | | | | |
| Q2 | 0.284 | 0.115 | 0.935 | 0.737 | 0.398 | 0.203 |
| Q3 | 0.288 | 0.253 | 0.339 | 0.242 | 0.31 | 0.351 |
| Q4 | 0.706 | 0.015^*^ | 0.337 | 0.288 | 0.732 | 0.047^*^ |
| Relative sugar intake | 0.494 | 0.205 | 0.268 | 0.013^*^ | 0.272 | 0.226 |
| Relative sugar intake, quartile |  |  |  |  |  |  |
| Q1 | Reference | | | | | |
| Q2 | 0.764 | 0.097 | 0.595 | 0.143 | 0.891 | 0.407 |
| Q3 | 0.647 | 0.249 | 0.395 | 0.044^*^ | 0.151 | 0.891 |
| Q4 | 0.398 | 0.411 | 0.399 | 0.032^*^ | 0.815 | 0.359 |
| High-sugar dietary score | 0.763 | 0.176 | 0.861 | 0.023^*^ | 0.393 | 0.465 |
| High-sugar dietary score, quartile |  |  |  |  |  |  |
| Q1 | Reference | | | | | |
| Q2 | 0.971 | 0.427 | 0.992 | 0.915 | 0.566 | 0.299 |
| Q3 | 0.488 | 0.322 | 0.334 | 0.44 | 0.773 | 0.214 |
| Q4 | 0.494 | 0.731 | 0.456 | 0.059 | 0.8 | 0.827 |

**^a^ All analyses were performed in fully adjusted Model 2.**

**^*^Statistically significant interaction effect was observed (p<0.05).**

# Table S13-P values on the interaction between exposures and stratification variables with Alzheimer’s disease incidents^a^

|  | **Alzheimer’s disease** | | | | | |
| --- | --- | --- | --- | --- | --- | --- |
|  | Age | Sex | BMI | APOE-state | Diabetes | Hypertension |
| Absolute sugar intake | 0.757 | 0.33 | 0.488 | 0.511 | 0.884 | 0.871 |
| Absolute sugar intake, quartile |  |  |  |  |  |  |
| Q1 | Reference | | | | | |
| Q2 | 0.737 | 0.639 | 0.105 | 0.233 | 0.413 | 0.725 |
| Q3 | 0.747 | 0.766 | 0.541 | 0.877 | 0.388 | 0.618 |
| Q4 | 0.862 | 0.321 | 0.873 | 0.674 | 0.645 | 0.655 |
| Relative sugar intake | 0.615 | 0.287 | 0.836 | 0.605 | 0.924 | 0.743 |
| Relative sugar intake, quartile |  |  |  |  |  |  |
| Q1 | Reference | | | | | |
| Q2 | 0.196 | 0.928 | 0.062 | 0.509 | 0.578 | 0.346 |
| Q3 | 0.108 | 0.51 | 0.588 | 0.537 | 0.934 | 0.588 |
| Q4 | 0.265 | 0.743 | 0.673 | 0.814 | 0.74 | 0.808 |
| High-sugar dietary score | 0.723 | 0.304 | 0.981 | 0.427 | 0.441 | 0.922 |
| High-sugar dietary score, quartile |  |  |  |  |  |  |
| Q1 | Reference | | | | | |
| Q2 | 0.502 | 0.749 | 0.626 | 0.741 | 0.97 | 0.457 |
| Q3 | 0.71 | 0.405 | 0.25 | 0.618 | 0.798 | 0.159 |
| Q4 | 0.624 | 0.94 | 0.741 | 0.615 | 0.61 | 0.796 |

**^a^ All analyses were performed in fully adjusted Model 2.**

**^*^Statistically significant interaction effect was observed (p<0.05).**

# Supplementary Table 13-Age-specific subgroup analyses of associations between sugar intake, high-sugar dietary score and all-cause dementia^a^

| All-cause dementia | Age≤55 | | | | Age 56-65 | | | | Age>65 | | | |
| --- | --- | --- | --- | --- | --- | --- | --- | --- | --- | --- | --- | --- |
|  | Number of events= 78 | | | | Number of events= 715 | | | | Number of events= 598 | | | |
|  | HR | 95%CI | | *P* | HR | 95%CI | | *P* | HR | 95%CI | | *P* |
| Absolute sugar intake | 1.003 | 0.999 | 1.007 | 0.135 | 1.004 | 1.002 | 1.005 | <0.001* | 1.001 | 1 | 1.003 | 0.119 |
| Absolute sugar intake, quartile |  | | | |  | | | |  | | | |
| Q1 | Reference | | | | Reference | | | | Reference | | | |
| Q2 | 0.523 | 0.263 | 1.037 | 0.064 | 0.893 | 0.716 | 1.114 | 0.316 | 0.85 | 0.666 | 1.085 | 0.191 |
| Q3 | 0.644 | 0.335 | 1.239 | 0.188 | 0.906 | 0.728 | 1.128 | 0.377 | 0.922 | 0.728 | 1.166 | 0.498 |
| Q4 | 1.248 | 0.719 | 2.163 | 0.431 | 1.285 | 1.048 | 1.575 | 0.016 | 1.048 | 0.837 | 1.311 | 0.685 |
| p for trend | 0.306 | | | | 0.004* | | | | 0.373 | | | |
| Relative sugar intake | 1.36 | 0.867 | 2.135 | 0.181 | 1.495 | 1.275 | 1.753 | <0.001* | 1.113 | 0.926 | 1.337 | 0.252 |
| Relative sugar intake, quartile |  | | | |  | | | |  | | | |
| Q1 | Reference | | | | Reference | | | | Reference | | | |
| Q2 | 1.064 | 0.576 | 1.965 | 0.844 | 1.056 | 0.841 | 1.325 | 0.641 | 0.851 | 0.667 | 1.086 | 0.194 |
| Q3 | 0.748 | 0.374 | 1.497 | 0.412 | 1.045 | 0.832 | 1.312 | 0.704 | 0.851 | 0.669 | 1.082 | 0.188 |
| Q4 | 1.349 | 0.748 | 2.433 | 0.32 | 1.564 | 1.268 | 1.93 | <0.001* | 1.076 | 0.858 | 1.35 | 0.526 |
| p for trend | 0.429 | | | | <0.001* | | | | 0.293 | | | |
| High-sugar dietary score | 1.093 | 0.929 | 1.287 | 0.284 | 1.12 | 1.058 | 1.187 | <0.001* | 1.05 | 0.984 | 1.122 | 0.142 |
| High-sugar dietary score,quartile |  | | | |  | | | |  | | | |
| Q1 | Reference | | | | Reference | | | | Reference | | | |
| Q2 | 1.056 | 0.55 | 2.026 | 0.87 | 0.914 | 0.731 | 1.142 | 0.429 | 0.885 | 0.689 | 1.137 | 0.338 |
| Q3 | 1.3 | 0.69 | 2.45 | 0.416 | 0.952 | 0.762 | 1.189 | 0.663 | 0.923 | 0.722 | 1.181 | 0.526 |
| Q4 | 1.297 | 0.682 | 2.466 | 0.428 | 1.292 | 1.047 | 1.594 | 0.017* | 1.19 | 0.941 | 1.505 | 0.147 |
| p for trend | 0.354 | | | | 0.007 | | | | 0.062 | | | |

**^a^ All analyses were performed in fully adjusted Model 2.**

**^*^Statistically significant interaction effect was observed (p<0.05).**

# Table S14-Sex-specific subgroup analyses of associations between sugar intake, high-sugar dietary score and all-cause dementia^a^

| ACD | Female | | | | Male | | | |
| --- | --- | --- | --- | --- | --- | --- | --- | --- |
|  | Number of events= 635 | | | | Number of events= 756 | | | |
|  | HR | 95%CI | | *P* | HR | 95%CI | | *P* |
| Absolute sugar intake | 1.004 | 1.003 | 1.005 | <0.001* | 1.001 | 1 | 1.003 | 0.035* |
| Absolute sugar intake, quartile |  | | | |  | | | |
| Q1 | Reference | | | | Reference | | | |
| Q2 | 0.966 | 0.768 | 1.215 | 0.767 | 0.749 | 0.601 | 0.934 | 0.010* |
| Q3 | 0.982 | 0.781 | 1.236 | 0.879 | 0.821 | 0.665 | 1.013 | 0.066 |
| Q4 | 1.425 | 1.148 | 1.768 | 0.001* | 0.991 | 0.815 | 1.205 | 0.926 |
| p for trend | 0.001* | | | | 0.434 | | | |
| Relative sugar intake | 1.42 | 1.208 | 1.668 | <0.001* | 1.22 | 1.033 | 1.441 | 0.019* |
| Relative sugar intake, quartile |  | | | |  | | | |
| Q1 | Reference | | | | Reference | | | |
| Q2 | 1.143 | 0.882 | 1.482 | 0.312 | 0.865 | 0.705 | 1.063 | 0.168 |
| Q3 | 1.066 | 0.825 | 1.377 | 0.625 | 0.877 | 0.712 | 1.081 | 0.219 |
| Q4 | 1.446 | 1.142 | 1.832 | 0.002* | 1.267 | 1.042 | 1.541 | 0.018* |
| p for trend | 0.001* | | | | 0.010* | | | |
| High-sugar dietary score | 1.128 | 1.058 | 1.202 | <0.001* | 1.063 | 1.006 | 1.123 | 0.030* |
| High-sugar dietary score,quartile |  | | | |  | | | |
| Q1 | Reference | | | | Reference | | | |
| Q2 | 1.003 | 0.748 | 1.345 | 0.983 | 0.868 | 0.712 | 1.059 | 0.162 |
| Q3 | 1.063 | 0.804 | 1.406 | 0.667 | 0.89 | 0.724 | 1.093 | 0.267 |
| Q4 | 1.251 | 0.956 | 1.639 | 0.103 | 1.319 | 1.091 | 1.596 | 0.004 |
| p for trend | 0.025* | | | | 0.01 | | | |

**^a^ All analyses were performed in fully adjusted Model 2.**

**^*^Statistically significant interaction effect was observed (p<0.05).**

# Table S15-APOE-specific subgroup analyses of associations between sugar intake, high-sugar dietary score and all-cause dementia^a^

| All-cause dementia | 0 APOE ε4 allele | | | | 1 APOE ε4 allele | | | | | 2 APOE ε4 allele | | | | |  |
| --- | --- | --- | --- | --- | --- | --- | --- | --- | --- | --- | --- | --- | --- | --- | --- |
|  | Number of events= 676 | | | | Number of events= 574 | | | | | Number of events= 141 | | | | |  |
|  | HR | 95%CI | | *P* | | HR | 95%CI | | *P* | | HR | 95%CI | | *P* | |
| Absolute sugar intake | 1.002 | 1 | 1.003 | 0.017* | | 1.004 | 1.002 | 1.005 | <0.001* | | 1.003 | 1 | 1.006 | 0.089 | |
| Absolute sugar intake, quartile |  | | | |  | | | | |  | | | | |  |
| Q1 | Reference | | | | Reference | | | | | Reference | | | | |  |
| Q2 | 0.865 | 0.695 | 1.077 | 0.196 | | 0.788 | 0.609 | 1.019 | 0.069 | | 1.036 | 0.616 | 1.743 | 0.893 | |
| Q3 | 0.797 | 0.638 | 0.996 | 0.046* | | 1.049 | 0.825 | 1.334 | 0.694 | | 0.81 | 0.484 | 1.357 | 0.424 | |
| Q4 | 1.089 | 0.886 | 1.338 | 0.418 | | 1.25 | 0.995 | 1.572 | 0.055 | | 1.298 | 0.803 | 2.1 | 0.287 | |
| p for trend | 0.311 | | | | 0.004* | | | | | 0.262 | | | | |  |
| Relative sugar intake | 1.139 | 0.961 | 1.351 | 0.133 | | 1.516 | 1.271 | 1.809 | <0.001* | | 1.431 | 1.001 | 2.046 | 0.049* | |
| Relative sugar intake, quartile |  | | | |  | | | | |  | | | | |  |
| Q1 | Reference | | | | Reference | | | | | Reference | | | | |  |
| Q2 | 0.831 | 0.666 | 1.037 | 0.102 | | 1.238 | 0.955 | 1.606 | 0.107 | | 0.776 | 0.443 | 1.357 | 0.374 | |
| Q3 | 0.83 | 0.664 | 1.036 | 0.099 | | 1.106 | 0.848 | 1.442 | 0.458 | | 1.037 | 0.622 | 1.73 | 0.89 | |
| Q4 | 1.132 | 0.921 | 1.39 | 0.239 | | 1.66 | 1.299 | 2.122 | <0.001* | | 1.259 | 0.768 | 2.064 | 0.361 | |
| p for trend | 0.136 | | | | <0.001* | | | | | 0.128 | | | | |  |
| High-sugar dietary score | 1.054 | 0.992 | 1.119 | 0.091 | | 1.121 | 1.051 | 1.197 | 0.001* | | 1.147 | 1.005 | 1.31 | 0.042* | |
| High-sugar dietary score,quartile |  | | | |  | | | | |  | | | | |  |
| Q1 | Reference | | | | Reference | | | | | Reference | | | | |  |
| Q2 | 0.933 | 0.746 | 1.166 | 0.541 | | 0.915 | 0.707 | 1.185 | 0.501 | | 0.816 | 0.47 | 1.419 | 0.471 | |
| Q3 | 0.965 | 0.772 | 1.205 | 0.752 | | 0.962 | 0.745 | 1.242 | 0.764 | | 0.995 | 0.584 | 1.697 | 0.987 | |
| Q4 | 1.152 | 0.927 | 1.43 | 0.201 | | 1.338 | 1.053 | 1.7 | 0.017* | | 1.384 | 0.839 | 2.283 | 0.203 | |
| p for trend | 0.157 | | | | 0.004* | | | | | 0.07 | | | | |  |

**^a^ All analyses were performed in fully adjusted Model 2.**

**^*^Statistically significant interaction effect was observed (p<0.05).**

# Table S16-Age-specific subgroup analyses of associations between sugar intake, high-sugar dietary score and Alzheimer’s disease^a^

| **AD** | **Age≤55** | | | | **Age 56-65** | | | | **Age>65** | | | |
| --- | --- | --- | --- | --- | --- | --- | --- | --- | --- | --- | --- | --- |
|  | **Number of events= 21** | | | | **Number of events= 299** | | | | **Number of events= 250** | | | |
|  | **HR** | **95%CI** | | ***P*** | **HR** | **95%CI** | | ***P*** | **HR** | **95%CI** | | ***P*** |
| Absolute sugar intake | 1 | 0.992 | 1.009 | 0.927 | 1.003 | 1.001 | 1.005 | 0.010^*^ | 1.002 | 0.999 | 1.004 | 0.145 |
| Absolute sugar intake, quartile |  | | | |  | | | |  | | | |
| Q1 | Reference | | | | Reference | | | | Reference | | | |
| Q2 | 0.246 | 0.053 | 1.144 | 0.074 | 1.102 | 0.781 | 1.556 | 0.58 | 0.814 | 0.553 | 1.198 | 0.296 |
| Q3 | 0.517 | 0.158 | 1.692 | 0.275 | 1.088 | 0.772 | 1.532 | 0.631 | 0.957 | 0.665 | 1.379 | 0.815 |
| Q4 | 0.836 | 0.294 | 2.379 | 0.737 | 1.339 | 0.963 | 1.863 | 0.083 | 1.101 | 0.778 | 1.558 | 0.586 |
| p for trend | 0.851 | | | | 0.078 | | | | 0.314 | | | |
| Relative sugar intake | 1.037 | 0.409 | 2.625 | 0.94 | 1.358 | 1.059 | 1.742 | 0.016^*^ | 1.144 | 0.863 | 1.517 | 0.349 |
| Relative sugar intake, quartile |  | | | |  | | | |  | | | |
| Q1 | Reference | | | | Reference | | | | Reference | | | |
| Q2 | 2.176 | 0.653 | 7.254 | 0.206 | 0.985 | 0.689 | 1.409 | 0.935 | 0.899 | 0.619 | 1.307 | 0.577 |
| Q3 | 1.132 | 0.282 | 4.551 | 0.862 | 1.104 | 0.782 | 1.56 | 0.573 | 0.746 | 0.508 | 1.096 | 0.136 |
| Q4 | 1.348 | 0.359 | 5.064 | 0.658 | 1.417 | 1.021 | 1.967 | 0.037^*^ | 1.125 | 0.793 | 1.594 | 0.51 |
| p for trend | 0.945 | | | | 0.015^*^ | | | | 0.423 | | | |
| High-sugar dietary score | 1.021 | 0.725 | 1.437 | 0.907 | 1.075 | 0.981 | 1.177 | 0.123 | 1.061 | 0.959 | 1.173 | 0.252 |
| High-sugar dietary score,quartile |  | | | |  | | | |  | | | |
| Q1 | Reference | | | | Reference | | | | Reference | | | |
| Q2 | 0.761 | 0.168 | 3.448 | 0.723 | 0.86 | 0.606 | 1.219 | 0.397 | 0.871 | 0.591 | 1.286 | 0.488 |
| Q3 | 2.323 | 0.694 | 7.772 | 0.171 | 0.953 | 0.678 | 1.341 | 0.783 | 0.888 | 0.604 | 1.306 | 0.546 |
| Q4 | 1.287 | 0.335 | 4.939 | 0.713 | 1.145 | 0.824 | 1.591 | 0.42 | 1.232 | 0.858 | 1.769 | 0.258 |
| p for trend | 0.434 | | | | 0.268 | | | | 0.148 | | | |

**^a^ All analyses were performed in fully adjusted Model 2.**

**^*^Statistically significant interaction effect was observed (p<0.05).**

# Table S17-Sex-specific subgroup analyses of associations between sugar intake, high-sugar dietary score and Alzheimer’s disease^a^

| **AD** | **Female** | | | | **Male** | | | |
| --- | --- | --- | --- | --- | --- | --- | --- | --- |
|  | **Number of events= 274** | | | | **Number of events= 296** | | | |
|  | **HR** | **95%CI** | | ***P*** | **HR** | **95%CI** | | ***P*** |
| Absolute sugar intake | 1.003 | 1.001 | 1.005 | 0.007^*^ | 1.001 | 0.999 | 1.004 | 0.197 |
| Absolute sugar intake, quartile |  | | | |  | | | |
| Q1 | Reference | | | | Reference | | | |
| Q2 | 0.966 | 0.68 | 1.373 | 0.848 | 0.856 | 0.599 | 1.224 | 0.394 |
| Q3 | 1.023 | 0.723 | 1.446 | 0.899 | 0.951 | 0.676 | 1.337 | 0.771 |
| Q4 | 1.345 | 0.964 | 1.876 | 0.081 | 1.059 | 0.766 | 1.464 | 0.728 |
| p for trend | 0.053 | | | | 0.455 | | | |
| Relative sugar intake | 1.376 | 1.075 | 1.763 | 0.011^*^ | 1.121 | 0.856 | 1.467 | 0.405 |
| Relative sugar intake, quartile |  | | | |  | | | |
| Q1 | Reference | | | | Reference | | | |
| Q2 | 0.968 | 0.648 | 1.447 | 0.874 | 0.995 | 0.719 | 1.376 | 0.975 |
| Q3 | 1.031 | 0.702 | 1.513 | 0.878 | 0.869 | 0.619 | 1.22 | 0.418 |
| Q4 | 1.35 | 0.946 | 1.926 | 0.098 | 1.235 | 0.898 | 1.701 | 0.195 |
| p for trend | 0.034^*^ | | | | 0.239 | | | |
| High-sugar dietary score | 1.11 | 1.005 | 1.225 | 0.039^*^ | 1.036 | 0.948 | 1.131 | 0.437 |
| High-sugar dietary score,quartile |  | | | |  | | | |
| Q1 | Reference | | | | Reference | | | |
| Q2 | 0.828 | 0.522 | 1.314 | 0.422 | 0.914 | 0.67 | 1.246 | 0.569 |
| Q3 | 1.074 | 0.703 | 1.642 | 0.741 | 0.864 | 0.621 | 1.202 | 0.385 |
| Q4 | 1.226 | 0.813 | 1.849 | 0.331 | 1.204 | 0.884 | 1.64 | 0.24 |
| p for trend | 0.061 | | | | 0.344 | | | |

**^a^ All analyses were performed in fully adjusted Model 2.**

**^*^Statistically significant interaction effect was observed (p<0.05).**

**Table S18-APOE-specific subgroup analyses of associations between sugar intake, high-sugar dietary score and Alzheimer’s disease^a^**

| **AD** | **0 APOE ε4 allele** | | | | **1 APOE ε4 allele** | | | | **2 APOE ε4 allele** | | | |
| --- | --- | --- | --- | --- | --- | --- | --- | --- | --- | --- | --- | --- |
|  | **Number of events= 212** | | | | **Number of events= 266** | | | | **Number of events= 92** | | | |
|  | **HR** | **95%CI** | | ***P*** | **HR** | **95%CI** | | ***P*** | **HR** | **95%CI** | | ***P*** |
| Absolute sugar intake | 1.002 | 1 | 1.005 | 0.09 | 1.003 | 1.001 | 1.005 | 0.012^*^ | 1 | 0.996 | 1.004 | 0.925 |
| Absolute sugar intake, quartile |  | | | |  | | | |  | | | |
| Q1 | Reference | | | | Reference | | | | Reference | | | |
| Q2 | 0.721 | 0.48 | 1.083 | 0.115 | 1.11 | 0.762 | 1.616 | 0.586 | 0.927 | 0.497 | 1.729 | 0.812 |
| Q3 | 0.842 | 0.572 | 1.24 | 0.383 | 1.316 | 0.915 | 1.892 | 0.138 | 0.652 | 0.345 | 1.23 | 0.187 |
| Q4 | 1.09 | 0.758 | 1.567 | 0.641 | 1.316 | 0.92 | 1.884 | 0.133 | 1.145 | 0.644 | 2.035 | 0.645 |
| p for trend | 0.359 | | | | 0.102 | | | | 0.602 | | | |
| Relative sugar intake | 1.102 | 0.812 | 1.497 | 0.533 | 1.477 | 1.139 | 1.915 | 0.003^*^ | 0.992 | 0.619 | 1.588 | 0.972 |
| Relative sugar intake, quartile |  | | | |  | | | |  | | | |
| Q1 | Reference | | | | Reference | | | | Reference | | | |
| Q2 | 0.969 | 0.657 | 1.43 | 0.875 | 1.22 | 0.824 | 1.806 | 0.321 | 0.567 | 0.297 | 1.081 | 0.085 |
| Q3 | 0.784 | 0.52 | 1.181 | 0.244 | 1.196 | 0.809 | 1.769 | 0.37 | 0.731 | 0.408 | 1.31 | 0.293 |
| Q4 | 1.183 | 0.816 | 1.715 | 0.376 | 1.69 | 1.172 | 2.438 | 0.005^*^ | 0.747 | 0.42 | 1.329 | 0.321 |
| p for trend | 0.423 | | | | 0.003^*^ | | | | 0.622 | | | |
| High-sugar dietary score | 1.03 | 0.924 | 1.148 | 0.595 | 1.118 | 1.015 | 1.231 | 0.023^*^ | 1.02 | 0.859 | 1.212 | 0.821 |
| High-sugar dietary score,quartile |  | | | |  | | | |  | | | |
| Q1 | Reference | | | | Reference | | | | Reference | | | |
| Q2 | 0.955 | 0.643 | 1.418 | 0.819 | 0.857 | 0.578 | 1.27 | 0.442 | 0.749 | 0.391 | 1.431 | 0.381 |
| Q3 | 0.932 | 0.626 | 1.388 | 0.728 | 1.053 | 0.723 | 1.532 | 0.788 | 0.852 | 0.454 | 1.599 | 0.618 |
| Q4 | 1.123 | 0.762 | 1.654 | 0.558 | 1.357 | 0.949 | 1.939 | 0.094 | 0.962 | 0.523 | 1.769 | 0.9 |
| p for trend | 0.554 | | | | 0.026^*^ | | | | 0.875 | | | |

**^a^ All analyses were performed in fully adjusted Model 2.**

**^*^Statistically significant interaction effect was observed (p<0.05).**

# Table S19-Mediation analyses of absolute total sugar intake and dementia

|  |  | **Indirect effect (Mediated effect)** | | | | **Percentage mediated** | | | | **Total effect** | | | | **Direct effect** | | | |
| --- | --- | --- | --- | --- | --- | --- | --- | --- | --- | --- | --- | --- | --- | --- | --- | --- | --- |
|  |  | **Effect**  **Estimate** | **95%CI** | | **p-value** | **Effect**  **Estimate** | **95%CI** | | **p-value** | **Effect**  **Estimate** | **95%CI** | | **p-value** | **Effect**  **Estimate** | **95%CI** | | **p-value** |
| ACD | **Inflammatory markers** |  |  |  |  |  |  |  |  |  |  |  |  |  |  |  |  |
|  | Absolute sugar intake-NLR-ACD | <0.001 | <0.001 | <0.001 | 0.002 | 0.007 | 0.003 | 0.013 | 0.005 | <0.001 | <0.001 | <0.001 | <0.001 | <0.001 | <0.001 | <0.001 | <0.001 |
|  | Absolute sugar intake-CRP-ACD | -0.001 | <-0.001 | -0.001 | 0.024 | -0.006 | -0.012 | -0.001 | 0.043 | <0.001 | <0.001 | <0.001 | <0.001 | <0.001 | <0.001 | <0.001 | <0.001 |
|  | **Vascular markers** |  |  |  |  |  |  |  |  |  |  |  |  |  |  |  |  |
|  | Absolute sugar intake-SBP-ACD | <0.001 | <0.001 | <0.001 | <0.001 | 0.04 | 0.028 | 0.059 | 0.008 | <0.001 | <0.001 | <0.001 | <0.001 | <0.001 | <0.001 | <0.001 | <0.001 |
|  | Absolute sugar intake-DBP-ACD | <0.001 | <0.001 | <0.001 | 0.126 | <0.001 | -0.001 | 0.001 | 0.729 | <0.001 | <0.001 | <0.001 | <0.001 | <0.001 | <0.001 | <0.001 | <0.001 |
|  | **Metabolic markers** |  |  |  |  |  |  |  |  |  |  |  |  |  |  |  |  |
|  | Absolute sugar intake-BMI-ACD | -0.001 | <-0.001 | -0.001 | 0.001 | -0.01 | -0.018 | -0.005 | 0.002 | <0.001 | <0.001 | <0.001 | <0.001 | <0.001 | <0.001 | <0.001 | <0.001 |
|  | Absolute sugar intake-HbA1c-ACD | -0.001 | <-0.001 | -0.001 | 0.001 | -0.017 | -0.029 | -0.007 | 0.004 | <0.001 | <0.001 | <0.001 | <0.001 | <0.001 | <0.001 | <0.001 | <0.001 |
| AD | **Inflammatory markers** |  |  |  |  |  |  |  |  |  |  |  |  |  |  |  |  |
|  | Absolute sugar intake-NLR-AD | <0.001 | <0.001 | <0.001 | 0.008 | 0.004 | 0.001 | 0.009 | 0.026 | <0.001 | <0.001 | <0.001 | <0.001 | <0.001 | <0.001 | <0.001 | <0.001 |
|  | Absolute sugar intake-CRP-AD | <0.001 | <0.001 | <0.001 | 0.294 | 0.003 | -0.003 | 0.009 | 0.043 | <0.001 | <0.001 | <0.001 | <0.001 | <0.001 | <0.001 | <0.001 | <0.001 |
|  | **Vascular markers** |  |  |  |  |  |  |  |  |  |  |  |  |  |  |  |  |
|  | Absolute sugar intake-SBP-AD | <0.001 | <0.001 | <0.001 | <0.001 | 0.041 | 0.027 | 0.069 | <0.001 | <0.001 | <0.001 | <0.001 | <0.001 | <0.001 | <0.001 | <0.001 | <0.001 |
|  | Absolute sugar intake-DBP-AD | <0.001 | <0.001 | <0.001 | <0.001 | <0.001 | -0.001 | 0.001 | 0.845 | <0.001 | <0.001 | <0.001 | <0.001 | <0.001 | <0.001 | <0.001 | <0.001 |
|  | **Metabolic markers** |  |  |  |  |  |  |  |  |  |  |  |  |  |  |  |  |
|  | Absolute sugar intake-BMI-AD | <0.001 | <0.001 | <0.001 | 0.6 | 0.002 | -0.005 | 0.009 | 0.619 | <0.001 | <0.001 | <0.001 | <0.001 | <0.001 | <0.001 | <0.001 | <0.001 |
|  | Absolute sugar intake-HbA1c-AD | -0.001 | <-0.001 | -0.001 | 0.002 | -0.014 | -0.026 | -0.006 | 0.008 | <0.001 | <0.001 | <0.001 | <0.001 | <0.001 | <0.001 | <0.001 | <0.001 |

# Table S20-Mediation analyses of relative total sugar intake and dementia

|  |  | **Indirect effect (Mediated effect)** | | | | **Percentage mediated** | | | | **Total effect** | | | | **Direct effect** | | | |
| --- | --- | --- | --- | --- | --- | --- | --- | --- | --- | --- | --- | --- | --- | --- | --- | --- | --- |
|  |  | **Effect**  **Estimate** | **95%CI** | | **p-value** | **Effect**  **Estimate** | **95%CI** | | **p-value** | **Effect**  **Estimate** | **95%CI** | | **p-value** | **Effect**  **Estimate** | **95%CI** | | **p-value** |
| ACD | **Inflammatory markers** |  |  |  |  |  |  |  |  |  |  |  |  |  |  |  |  |
|  | Relative sugar intake -NLR-ACD | <-0.001 | <-0.001 | <-0.001 | <0.001 | -0.014 | -0.022 | -0.008 | <0.001 | 0.004 | 0.003 0.005 | | <0.001 | 0.004 | 0.003 0.005 | | <0.001 |
|  | Relative sugar intake-CRP-ACD | <-0.001 | <-0.001 | <-0.001 | 0.195 | -0.001 | -0.004 | <0.001 | 0.215 | 0.004 | 0.003 0.005 | | <0.001 | 0.004 | 0.003 0.005 | | <0.001 |
|  | **Vascular markers** |  |  |  |  |  |  |  |  |  |  |  |  |  |  |  |  |
|  | Relative sugar intake-SBP-ACD | <-0.001 | <-0.001 | <-0.001 | <0.001 | -0.009 | -0.019 | -0.001 | 0.043 | 0.004 | 0.003 0.005 | | <0.001 | 0.004 | 0.003 0.005 | | <0.001 |
|  | Relative sugar intake-DBP-ACD | <0.001 | <0.001 | <0.001 | 0.386 | 0.004 | -0.006 | 0.014 | 0.398 | 0.004 | 0.003 0.005 | | <0.001 | 0.004 | 0.003 0.005 | | <0.001 |
|  | **Metabolic markers** |  |  |  |  |  |  |  |  |  |  |  |  |  |  |  |  |
|  | Relative sugar intake-BMI-ACD | <-0.001 | <-0.001 | <-0.001 | <0.001 | -0.025 | -0.04 | -0.013 | <0.001 | 0.004 | 0.003 0.005 | | <0.001 | 0.004 | 0.003 0.005 | | <0.001 |
|  | Relative sugar intake-HbA1c-ACD | <-0.001 | <-0.001 | <-0.001 | <0.001 | -0.044 | -0.066 | -0.03 | <0.001 | 0.004 | 0.003 0.005 | | <0.001 | 0.004 | 0.003 0.005 | | <0.001 |
| AD | **Inflammatory markers** |  |  |  |  |  |  |  |  |  |  |  |  |  |  |  |  |
|  | Relative sugar intake-NLR-AD | <-0.001 | <-0.001 | <-0.001 | 0.002 | -0.008 | -0.015 | -0.003 | 0.011 | 0.002 | 0.001 | 0.003 | <0.001 | 0.002 | 0.001 | 0.003 | <0.001 |
|  | Relative sugar intake-CRP-AD | <-0.001 | <-0.001 | <-0.001 | 0.4 | 0.001 | -0.001 | 0.003 | 0.411 | 0.002 | 0.001 | 0.003 | <0.001 | 0.002 | 0.001 | 0.003 | <0.001 |
|  | **Vascular markers** |  |  |  |  |  |  |  |  |  |  |  |  |  |  |  |  |
|  | Relative sugar intake-SBP-AD | <-0.001 | <-0.001 | <-0.001 | 0.029 | -0.009 | -0.02 | -0.001 | 0.062 | 0.002 | 0.001 | 0.003 | <0.001 | 0.002 | 0.001 | 0.003 | <0.001 |
|  | Relative sugar intake-DBP-AD | <0.001 | <0.001 | <0.001 | 0.801 | 0.001 | -0.011 | 0.013 | 0.815 | 0.002 | 0.001 | 0.003 | <0.001 | 0.002 | 0.001 | 0.003 | <0.001 |
|  | **Metabolic markers** |  |  |  |  |  |  |  |  |  |  |  |  |  |  |  |  |
|  | Relative sugar intake-BMI-AD | <-0.001 | <-0.001 | <-0.001 | 0.757 | 0.002 | -0.012 | 0.018 | 0.766 | 0.002 | 0.001 | 0.003 | <0.001 | 0.002 | 0.001 | 0.003 | <0.001 |
|  | Relative sugar intake-HbA1c-AD | <-0.001 | <-0.001 | <-0.001 | <0.001 | -0.034 | -0.057 | -0.022 | <0.001 | 0.002 | 0.001 | 0.003 | <0.001 | 0.002 | 0.001 | 0.003 | <0.001 |

# Table S21-Sensitivity analysis excluding participants developing all-cause dementia within 3 years ^a^

|  | **ACD** | | | | **AD** | | | |
| --- | --- | --- | --- | --- | --- | --- | --- | --- |
|  | Number of events= 1314 | | | | Number of events= 532 | | | |
|  | HR | 95%CI | | P-value | HR | 95%CI | | P-value |
| **Absolute total sugar intake** | 1.003 | 1.002 | 1.004 | <0.001^*^ | 1.002 | 1.001 | 1.004 | 0.006^*^ |
| **Absolute sugar intake, quartile** |  | | | |  | | | |
| Q1 | Reference | | | | Reference | | | |
| Q2 | 0.871 | 0.74 | 1.026 | 0.098 | 0.914 | 0.708 | 1.181 | 0.493 |
| Q3 | 0.92 | 0.784 | 1.08 | 0.309 | 1.015 | 0.793 | 1.299 | 0.907 |
| Q4 | 1.183 | 1.018 | 1.375 | 0.028^*^ | 1.181 | 0.932 | 1.498 | 0.169 |
| **p for trend** | 0.004^*^ | | | | 0.072 | | | |
| **Relative total sugar intake** | 1.325 | 1.176 | 1.493 | <0.001^*^ | 1.278 | 1.062 | 1.539 | 0.010^*^ |
| **Relative sugar intake, quartile** |  | | | |  | | | |
| Q1 | Reference | | | | Reference | | | |
| Q2 | 0.996 | 0.845 | 1.175 | 0.963 | 1 | 0.771 | 1.296 | 0.998 |
| Q3 | 0.957 | 0.811 | 1.13 | 0.606 | 0.978 | 0.756 | 1.267 | 0.868 |
| Q4 | 1.339 | 1.148 | 1.562 | <0.001^*^ | 1.329 | 1.043 | 1.692 | 0.021^*^ |
| **p for trend** | <0.001^*^ | | | | 0.010^*^ | | | |
| **High-sugar dietary score** | 1.098 | 1.051 | 1.146 | <0.001^*^ | 1.075 | 1.005 | 1.15 | 0.036^*^ |
| **High-sugar dietary score，quartile** |  | | | |  | | | |
| Q1 | Reference | | | | Reference | | | |
| Q2 | 0.95 | 0.804 | 1.122 | 0.543 | 0.895 | 0.688 | 1.163 | 0.405 |
| Q3 | 0.98 | 0.831 | 1.157 | 0.815 | 0.994 | 0.769 | 1.284 | 0.962 |
| Q4 | 1.292 | 1.104 | 1.512 | 0.001^*^ | 1.231 | 0.963 | 1.574 | 0.097 |
| **p for trend** | <0.001^*^ | | | | 0.036^*^ | | | |

**^a^Analysis was performed in fully adjusted Model 3. ^*^Statistically significant difference was found (p<0.05).Abbreviation: HR, Hazard ratios; 95%CI, Confidential intervals**

# Table S22-Factor loadings for high-sugar dietary pattern among participants who completed at least two dietary assessments

| Food groups | Factor loadings | Food groups | Factor loadings |
| --- | --- | --- | --- |
| Fresh fruit | 0.612 | Alcoholic drinks(Wine, beer, spirits) | -0.369 |
| Sugar-sweetened beverages & other sugary drinks | 0.341 | High-fiber bread | -0.258 |
| Fruit juice | 0.336 | Low-fiber bread | -0.258 |
| Dried and stewed fruit | 0.284 | Pizza | -0.200 |
| Table sugars & preserves | 0.188 | Red meat | -0.194 |
| Chocolate and confectionery | 0.155 | Pasta and rice | -0.182 |
| Milk-based and powdered drinks | 0.153 | Other bread products | -0.159 |
| Low-fat milk | 0.140 | Fried or roast potatoes | -0.156 |
| Vegetables | 0.137 | Egg & egg dishes | -0.129 |
| Non-dairy milk | 0.031 | Poultry | -0.124 |
| Milk-based desserts | 0.030 | High fat cheese | -0.123 |
| Water/Sparkling water | 0.027 | Butter and other animal fat spreads | -0.122 |
| High-fat milk and cream | 0.026 | Crisps and savoury snacks | -0.117 |
| Sauces & condiments (low fat) | 0.017 | Processed meat | -0.112 |
| High-fiber breakfast cereals | 0.006 | Nuts & seeds | -0.110 |
| Coffee and tea | 0.003 | Coated or breaded meat and fish | -0.102 |
|  |  | Boiled or baked potatoes | -0.102 |
|  |  | Oily fish | -0.090 |
|  |  | Wholemeal pasta, brown rice & other wholegrains | -0.061 |
|  |  | Biscuits | -0.057 |
|  |  | Other fish | -0.056 |
|  |  | Normal plant-based fat spread | -0.056 |
|  |  | Meat substitutes | -0.054 |
|  |  | Lower plant-based fat spread | -0.039 |
|  |  | Low animal fat spread | -0.030 |
|  |  | Sauces & condiments (high fat) | -0.028 |
|  |  | Olive oil | -0.023 |
|  |  | Vegetable side dishes and dips | -0.022 |
|  |  | Grain-based desserts | -0.016 |
|  |  | Low fat cheese | -0.016 |
|  |  | Soups | -0.015 |
|  |  | Other breakfast cereals | -0.013 |
|  |  | Nut-based spread | -0.011 |
|  |  | Low/non sugar SSBs | -0.002 |
|  |  | Legumes & pulses | 0.000 |

# Table S23-Factor loadings for high-sugar dietary pattern among participants who completed at least three dietary assessments

| Food groups | Factor loadings | Food groups | Factor loadings |
| --- | --- | --- | --- |
| Fresh fruit | 0.618 | Alcoholic drinks(Wine, beer, spirits) | -0.379 |
| Fruit juice | 0.339 | High-fiber bread | -0.261 |
| Sugar-sweetened beverages & other sugary drinks | 0.331 | Low-fiber bread | -0.254 |
| Dried and stewed fruit | 0.285 | Pizza | -0.190 |
| Table sugars & preserves | 0.188 | Red meat | -0.188 |
| Chocolate and confectionery | 0.155 | Pasta and rice | -0.173 |
| Milk-based and powdered drinks | 0.142 | Other bread products | -0.152 |
| Low-fat milk | 0.141 | Fried or roast potatoes | -0.147 |
| Vegetables | 0.140 | Butter and other animal fat spreads | -0.123 |
| Non-dairy milk | 0.034 | Egg & egg dishes | -0.122 |
| Milk-based desserts | 0.030 | High fat cheese | -0.122 |
| Water/Sparkling water | 0.027 | Poultry | -0.114 |
| High-fat milk and cream | 0.027 | Crisps and savoury snacks | -0.111 |
| Sauces & condiments (low fat) | 0.017 | Processed meat | -0.108 |
| High-fiber breakfast cereals | 0.002 | Nuts & seeds | -0.107 |
| Legumes & pulses | 0.002 | Boiled or baked potatoes | -0.098 |
| Coffee and tea | 0.001 | Coated or breaded meat and fish | -0.093 |
|  |  | Oily fish | -0.086 |
|  |  | Wholemeal pasta, brown rice & other wholegrains | -0.060 |
|  |  | Biscuits | -0.056 |
|  |  | Normal plant-based fat spread | -0.056 |
|  |  | Other fish | -0.055 |
|  |  | Meat substitutes | -0.054 |
|  |  | Lower plant-based fat spread | -0.037 |
|  |  | Low animal fat spread | -0.029 |
|  |  | Sauces & condiments (high fat) | -0.026 |
|  |  | Olive oil | -0.023 |
|  |  | Vegetable side dishes and dips | -0.019 |
|  |  | Soups | -0.015 |
|  |  | Other breakfast cereals | -0.014 |
|  |  | Grain-based desserts | -0.012 |
|  |  | Nut-based spread | -0.011 |
|  |  | Low fat cheese | -0.011 |
|  |  | Low/non sugar SSBs | -0.002 |

# Table S24-Factor loadings for high-sugar dietary pattern among participants who completed at least four dietary assessments

| **Food groups** | **Factor loadings** | **Food groups** | **Factor loadings** |
| --- | --- | --- | --- |
| Fresh fruit | 0.620 | Alcoholic drinks(Wine, beer, spirits) | -0.384 |
| Fruit juice | 0.340 | High-fiber bread | -0.268 |
| Sugar-sweetened beverages & other sugary drinks | 0.323 | Low-fiber bread | -0.256 |
| Dried and stewed fruit | 0.292 | Pizza | -0.189 |
| Table sugars & preserves | 0.191 | Red meat | -0.185 |
| Chocolate and confectionery | 0.155 | Pasta and rice | -0.163 |
| Low-fat milk | 0.146 | Other bread products | -0.146 |
| Vegetables | 0.138 | Fried or roast potatoes | -0.139 |
| Milk-based and powdered drinks | 0.132 | Butter and other animal fat spreads | -0.121 |
| Non-dairy milk | 0.031 | High fat cheese | -0.120 |
| Milk-based desserts | 0.030 | Egg & egg dishes | -0.119 |
| High-fat milk and cream | 0.027 | Crisps and savoury snacks | -0.107 |
| Water/Sparkling water | 0.026 | Nuts & seeds | -0.103 |
| Sauces & condiments (low fat) | 0.015 | Poultry | -0.103 |
| Legumes & pulses | 0.000 | Processed meat | -0.103 |
|  |  | Boiled or baked potatoes | -0.098 |
|  |  | Coated or breaded meat and fish | -0.090 |
|  |  | Oily fish | -0.087 |
|  |  | Biscuits | -0.058 |
|  |  | Wholemeal pasta, brown rice & other whole grains | -0.056 |
|  |  | Normal plant-based fat spread | -0.054 |
|  |  | Meat substitutes | -0.049 |
|  |  | Other fish | -0.048 |
|  |  | Lower plant-based fat spread | -0.034 |
|  |  | Low animal fat spread | -0.030 |
|  |  | Sauces & condiments (high fat) | -0.027 |
|  |  | Olive oil | -0.024 |
|  |  | Vegetable side dishes and dips | -0.020 |
|  |  | Low fat cheese | -0.014 |
|  |  | Other breakfast cereals | -0.013 |
|  |  | Grain-based desserts | -0.013 |
|  |  | Soups | -0.012 |
|  |  | Nut-based spread | -0.010 |
|  |  | Coffee and tea | -0.003 |
|  |  | Low/non sugar SSBs | -0.003 |
|  |  | High-fiber breakfast cereals | -0.001 |

# Table S25-Sensitivity analysis among participants who completed at least two dietary assessments ^a^

|  | **ACD** | | | | **AD** | | | |
| --- | --- | --- | --- | --- | --- | --- | --- | --- |
|  | Number of events= 689 | | | | Number of events= 291 | | | |
|  | HR | 95%CI | | P-value | HR | 95%CI | | P-value |
| **Absolute sugar intake** | 1.004 | 1.002 | 1.006 | <0.001^*^ | 1.003 | 1 | 1.005 | 0.027^*^ |
| **Absolute sugar intake, quartile** |  | | | |  | | | |
| Q1 | Reference | | | | Reference | | | |
| Q2 | 0.944 | 0.744 | 1.198 | 0.637 | 0.949 | 0.661 | 1.363 | 0.777 |
| Q3 | 1.054 | 0.836 | 1.327 | 0.657 | 0.999 | 0.702 | 1.423 | 0.997 |
| Q4 | 1.414 | 1.129 | 1.77 | 0.003^*^ | 1.246 | 0.88 | 1.764 | 0.215 |
| **p for trend** | <0.001^*^ | | | | 0.115 | | | |
| **Relative sugar intake** | 1.601 | 1.324 | 1.937 | <0.001^*^ | 1.307 | 0.968 | 1.763 | 0.08 |
| **Relative sugar intake, quartile** |  | | | |  | | | |
| Q1 | Reference | | | | Reference | | | |
| Q2 | 1.232 | 0.968 | 1.568 | 0.09 | 1.09 | 0.752 | 1.578 | 0.65 |
| Q3 | 1.255 | 0.988 | 1.596 | 0.063 | 1.17 | 0.814 | 1.682 | 0.397 |
| Q4 | 1.641 | 1.295 | 2.079 | <0.001^*^ | 1.398 | 0.973 | 2.009 | 0.07 |
| **p for trend** | <0.001^*^ | | | | 0.046^*^ | | | |
| **High-sugar dietary score** | 1.116 | 1.053 | 1.183 | <0.001^*^ | 1.038 | 0.947 | 1.137 | 0.423 |
| **High-sugar dietary score，quartile** |  | | | |  | | | |
| Q1 | Reference | | | | Reference | | | |
| Q2 | 1.021 | 0.806 | 1.294 | 0.865 | 0.848 | 0.591 | 1.216 | 0.369 |
| Q3 | 1.085 | 0.859 | 1.371 | 0.495 | 0.984 | 0.694 | 1.394 | 0.926 |
| Q4 | 1.452 | 1.162 | 1.815 | 0.001^*^ | 1.137 | 0.81 | 1.597 | 0.458 |
| **p for trend** | <0.001^*^ | | | | 0.264 | | | |

**^a^Analysis was performed in fully adjusted Model 3. ^*^Statistically significant difference was found (p<0.05). Abbreviation: HR, Hazard ratios; 95%CI, Confidential intervals**

# Table S26-Sensitivity analysis among participants who completed at least three dietary assessments ^a^

|  | **ACD** | | | | **AD** | | | |
| --- | --- | --- | --- | --- | --- | --- | --- | --- |
|  | **Number of events= 386** | | | | **Number of events= 164** | | | |
|  | HR | 95%CI | | P-value | HR | 95%CI | | P-value |
| **Absolute sugar intake** | 1.004 | 1.001 | 1.006 | 0.002* | 1.002 | 0.998 | 1.005 | 0.377 |
| **Absolute sugar intake, quartile** |  | | | |  | | | |
| Q1 | Reference | | | | Reference | | | |
| Q2 | 0.946 | 0.687 | 1.303 | 0.734 | 1.229 | 0.749 | 2.017 | 0.414 |
| Q3 | 1.105 | 0.812 | 1.502 | 0.526 | 1.157 | 0.707 | 1.895 | 0.562 |
| Q4 | 1.348 | 0.991 | 1.833 | 0.057 | 1.324 | 0.804 | 2.179 | 0.27 |
| **p for trend** | 0.014* | | | | 0.345 | | | |
| **Relative sugar intake** | 1.766 | 1.354 | 2.305 | <0.001* | 1.326 | 0.87 | 2.021 | 0.189 |
| **Relative sugar intake, quartile** |  | | | |  | | | |
| Q1 | Reference | | | | Reference | | | |
| Q2 | 1.296 | 0.938 | 1.791 | 0.116 | 1.407 | 0.857 | 2.309 | 0.177 |
| Q3 | 1.262 | 0.912 | 1.746 | 0.16 | 1.117 | 0.67 | 1.861 | 0.672 |
| Q4 | 1.748 | 1.266 | 2.414 | 0.001* | 1.591 | 0.96 | 2.638 | 0.072 |
| **p for trend** | 0.001* | | | | 0.142 | | | |
| **High-sugar dietary score** | 1.11 | 1.027 | 1.199 | 0.008* | 1.007 | 0.891 | 1.138 | 0.91 |
| **High-sugar dietary score，quartile** |  | | | |  | | | |
| Q1 | Reference | | | | Reference | | | |
| Q2 | 1.283 | 0.937 | 1.755 | 0.12 | 1.057 | 0.656 | 1.704 | 0.819 |
| Q3 | 1.146 | 0.829 | 1.583 | 0.41 | 1.083 | 0.672 | 1.748 | 0.743 |
| Q4 | 1.652 | 1.217 | 2.242 | 0.001* | 1.264 | 0.792 | 2.016 | 0.326 |
| **p for trend** | 0.002* | | | | 0.301 | | | |

**^a^Analysis was performed in fully adjusted Model 3. ^*^Statistically significant difference was found (p<0.05). Abbreviation: HR, Hazard ratios; 95%CI, Confidential intervals**

# Table S27-Sensitivity analysis among participants who completed at least four dietary assessments ^a^

|  | **ACD** | | | | **AD** | | | |
| --- | --- | --- | --- | --- | --- | --- | --- | --- |
|  | **Number of events= 172** | | | | **Number of events= 71** | | | |
|  | HR | 95%CI | | P-value | HR | 95%CI | | P-value |
| **Absolute total sugar intake** | 1.005 | 1.001 | 1.008 | 0.005* | 1.001 | 0.996 | 1.007 | 0.66 |
| **Absolute total sugar intake, quartile** |  | | | |  | | | |
| Q1 | Reference | | | | Reference | | | |
| Q2 | 1.33 | 0.785 | 2.253 | 0.289 | 2.076 | 0.935 | 4.611 | 0.073 |
| Q3 | 1.708 | 1.032 | 2.829 | 0.037* | 1.402 | 0.61 | 3.222 | 0.426 |
| Q4 | 1.974 | 1.183 | 3.294 | 0.009* | 1.814 | 0.788 | 4.177 | 0.162 |
| **p for trend** | 0.004* | | | | 0.45 | | | |
| **Relative total sugar intake** | 2.196 | 1.477 | 3.265 | <0.001* | 1.422 | 0.748 | 2.703 | 0.283 |
| **Relative total sugar intake, quartile** |  | | | |  | | | |
| Q1 | Reference | | | | Reference | | | |
| Q2 | 1.726 | 1.007 | 2.958 | 0.047* | 1.813 | 0.799 | 4.115 | 0.155 |
| Q3 | 2.126 | 1.26 | 3.59 | 0.005* | 1.691 | 0.742 | 3.854 | 0.211 |
| Q4 | 2.469 | 1.442 | 4.227 | 0.001* | 2.212 | 0.963 | 5.083 | 0.061 |
| **p for trend** | 0.001* | | | | 0.094 | | | |
| **High-sugar dietary score** | 1.134 | 1.013 | 1.27 | 0.029* | 1.033 | 0.862 | 1.238 | 0.724 |
| **High-sugar dietary score, quartile** |  | | | |  | | | |
| Q1 | Reference | | | | Reference | | | |
| Q2 | 1.437 | 0.887 | 2.33 | 0.141 | 0.612 | 0.281 | 1.33 | 0.215 |
| Q3 | 1.486 | 0.916 | 2.411 | 0.108 | 1.069 | 0.539 | 2.12 | 0.848 |
| Q4 | 1.846 | 1.152 | 2.959 | 0.011* | 1.226 | 0.626 | 2.401 | 0.553 |
| **p for trend** | 0.012* | | | | 0.293 | | | |

**^a^Analysis was performed in fully adjusted model 3. ^*^Statistically significant difference was found (p<0.05). Abbreviation: HR, Hazard ratios; 95%CI, Confidential intervals**
